# Supplementary material for: Preparation of High Molecular Weight Poly(urethane-urea)s Bearing Deactivated Diamines
Source: Polymers (Basel). 2021 Jun 9;13(12):1914. doi: 10.3390/polym13121914 (PMC8229936; doi:10.3390/polym13121914)
Supplement: Supplementary file 1 [file polymers-13-01914-s001.zip › polymers-1218004-supplementary.pdf]

Supporting Information

Preparation of High Molecular Weight Poly(urethane-urea)s Bearing Deactivated Diamines

Alejandra Rubio Hernández-Sampelayo [1,2], Rodrigo Navarro [1,3,*] and Ángel Marcos-Fernández [1,3]

[1] Institute of Polymer Science and Technology (ICTP-CSIC) Juan de la Cierva 3, 28006Madrid, Spain; alerubioh@hotmail.com (A.R.H.-S.); amarcos@ictp.csic.es (Á.M.-F.)
[2] Universidad Nacional de Educación a Distancia (UNED), C/Bravo Murillo, 38, 28015 Madrid, Spain
[3] Interdisciplinary Platform for "Sustainable Plastics towards a Circular Economy" (SUS-PLAST-CSIC), Madrid, Spain
* Correspondence: rnavarro@ictp.csic.es; Tel.: +34-915622900

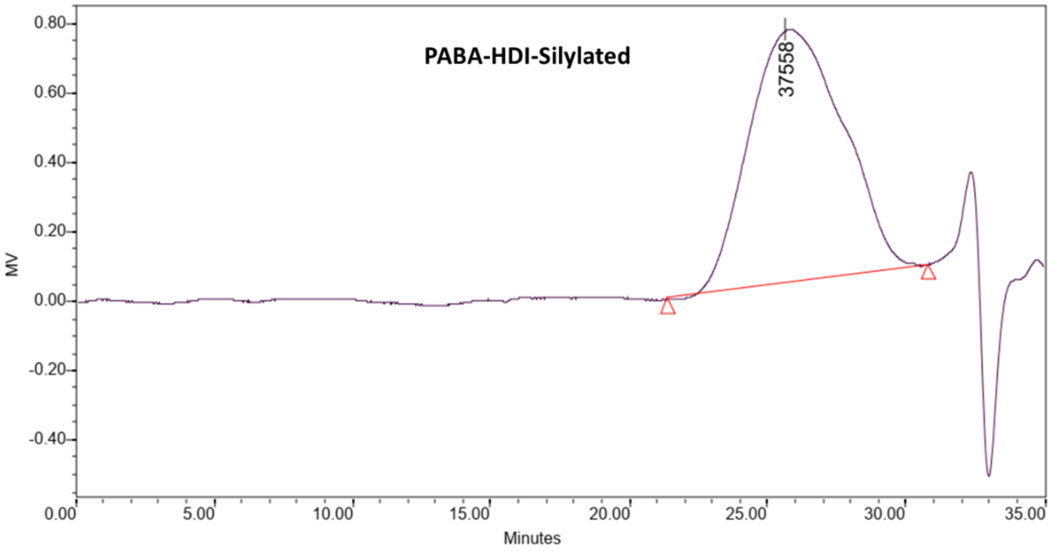

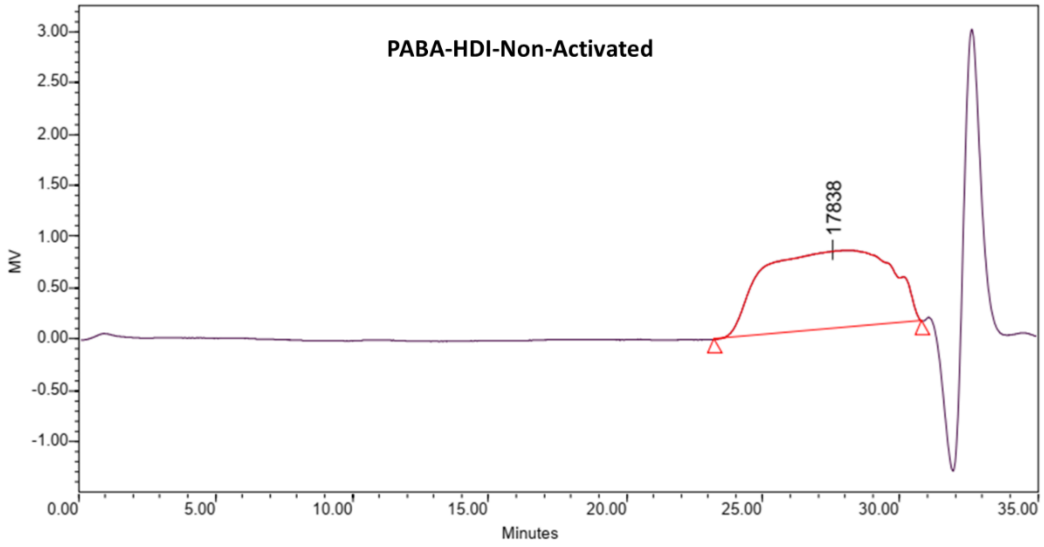

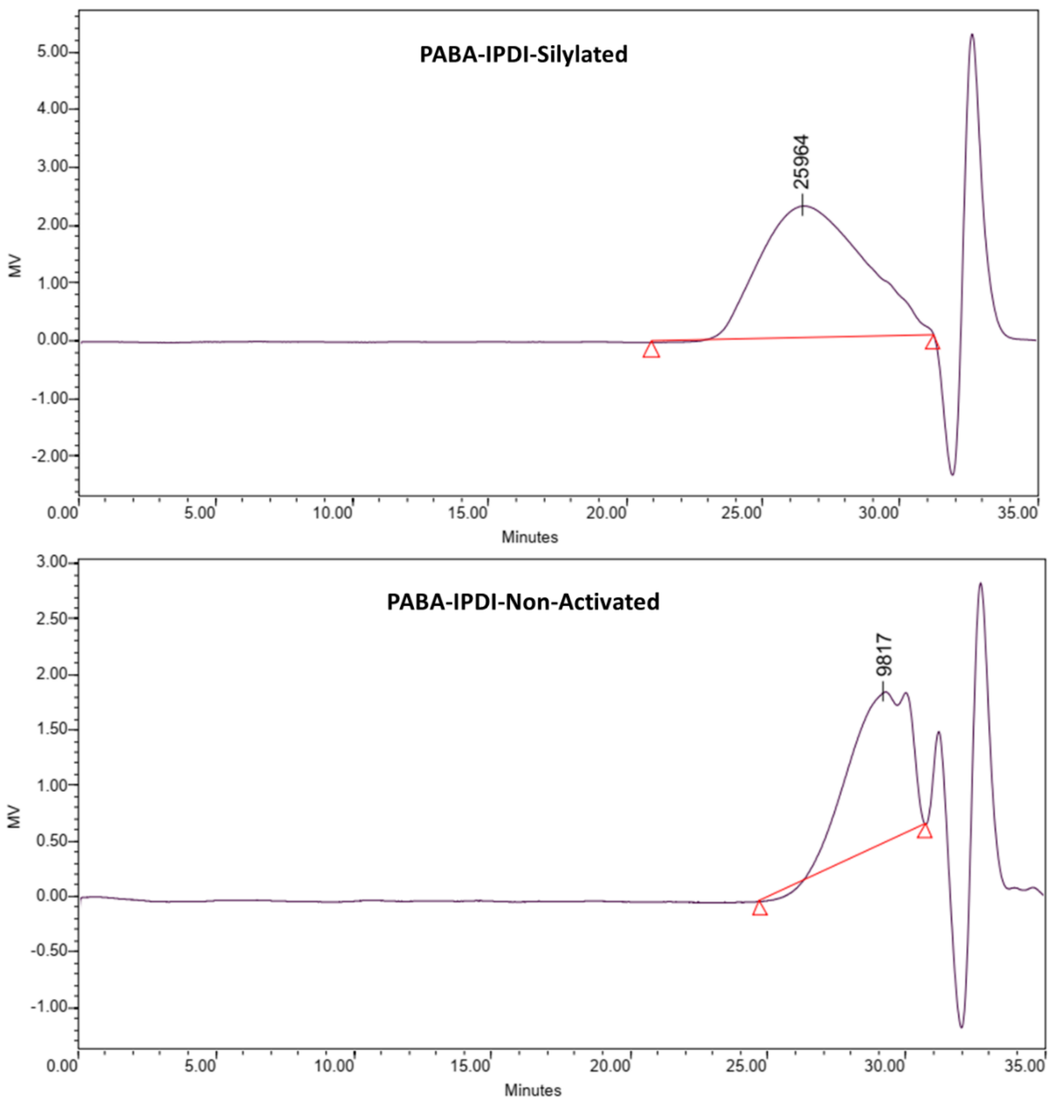

Figure S1. GPC chromatograms of synthetized Polyureas by silylation method and standard conditions.

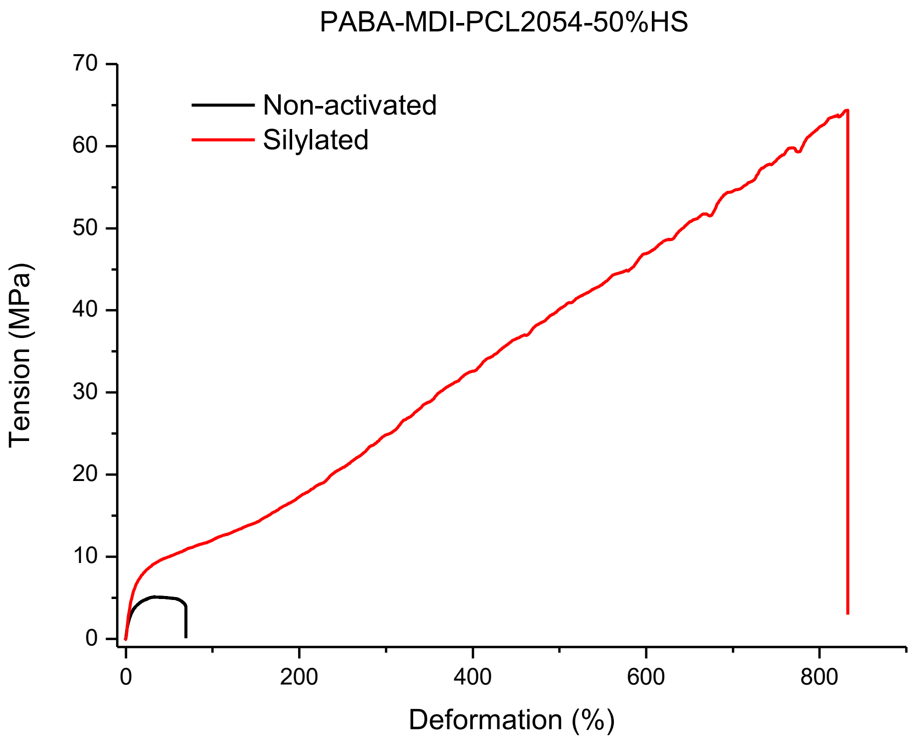

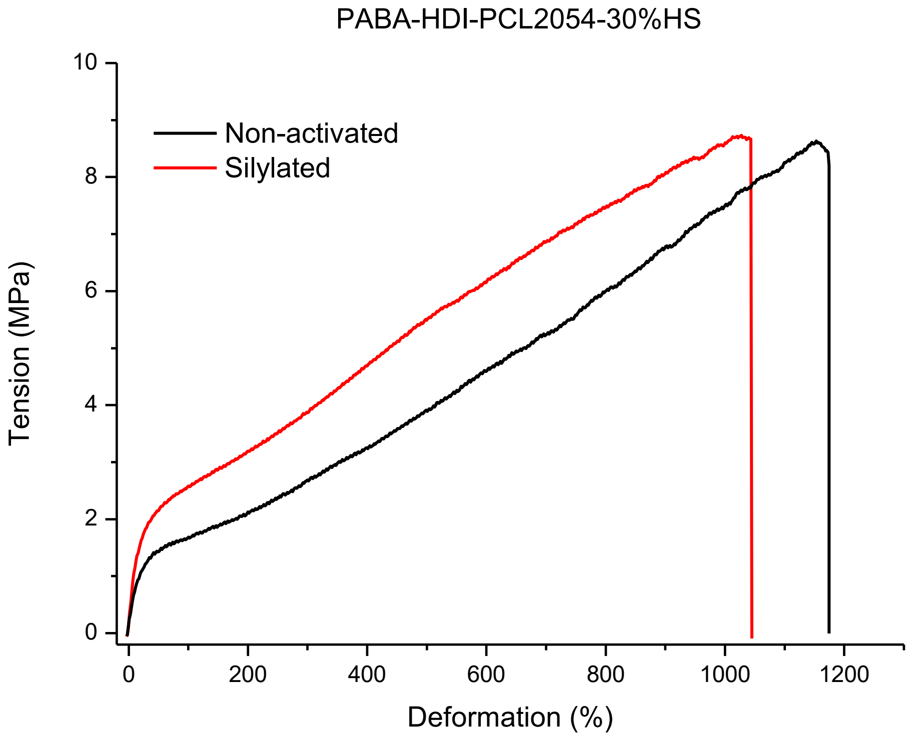

Figure S2. The stress-strain curves of silylated and non-silylated polymers PABA-MDI-PCL2054-50HS and PABA-HDI-PCL2054-30HS.

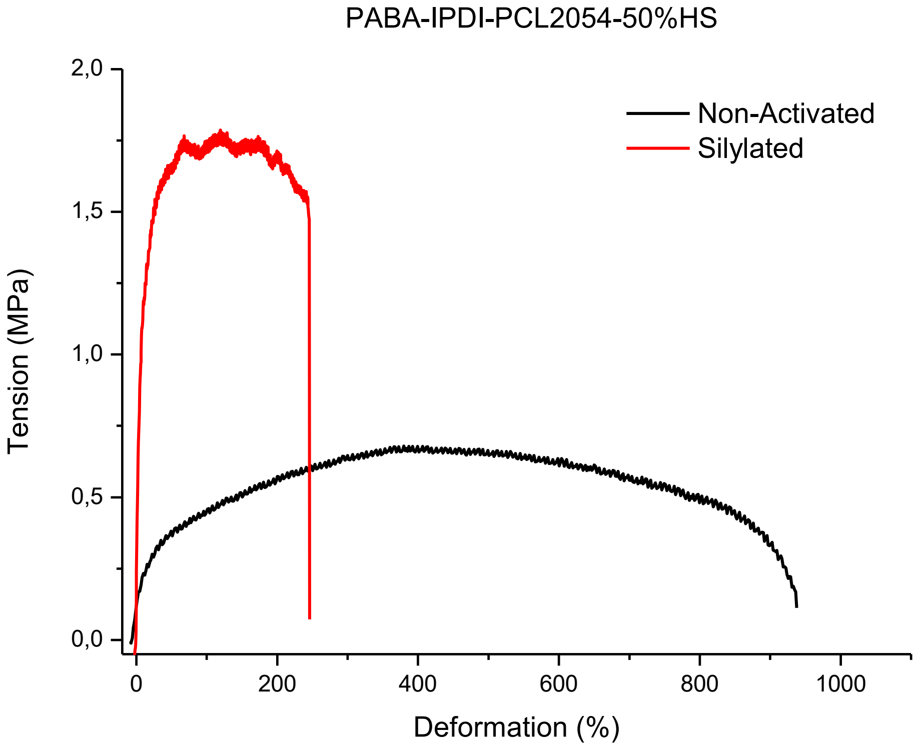

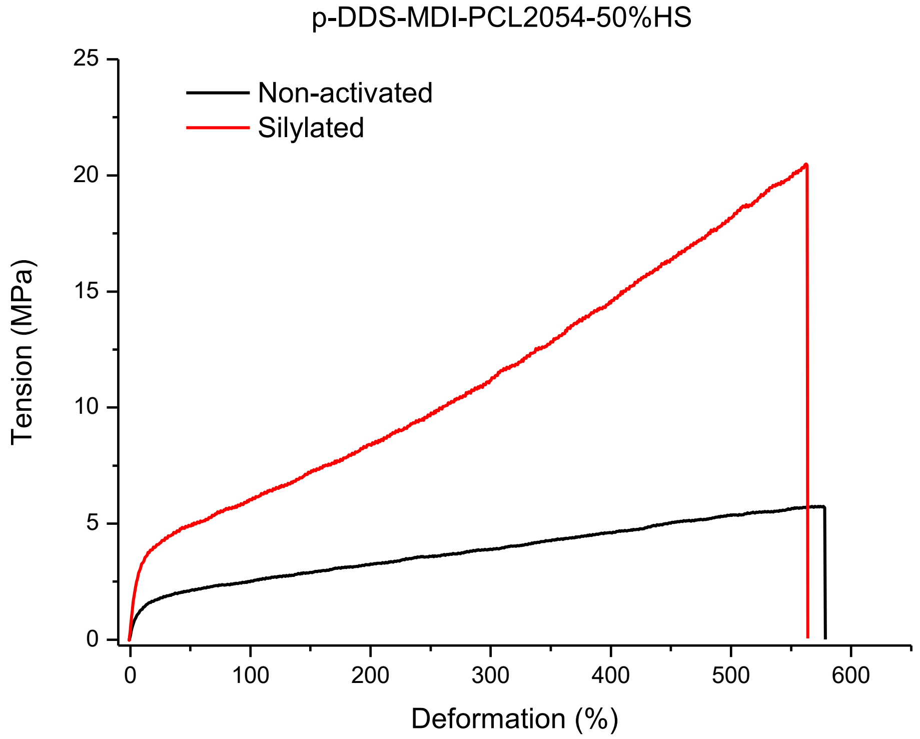

Figure S3. The stress-strain curves of silylated and non-silylated polymers PABA-IPDI-PCL2054-50HS and p-DDS-MDI-PCL2054-50HS.

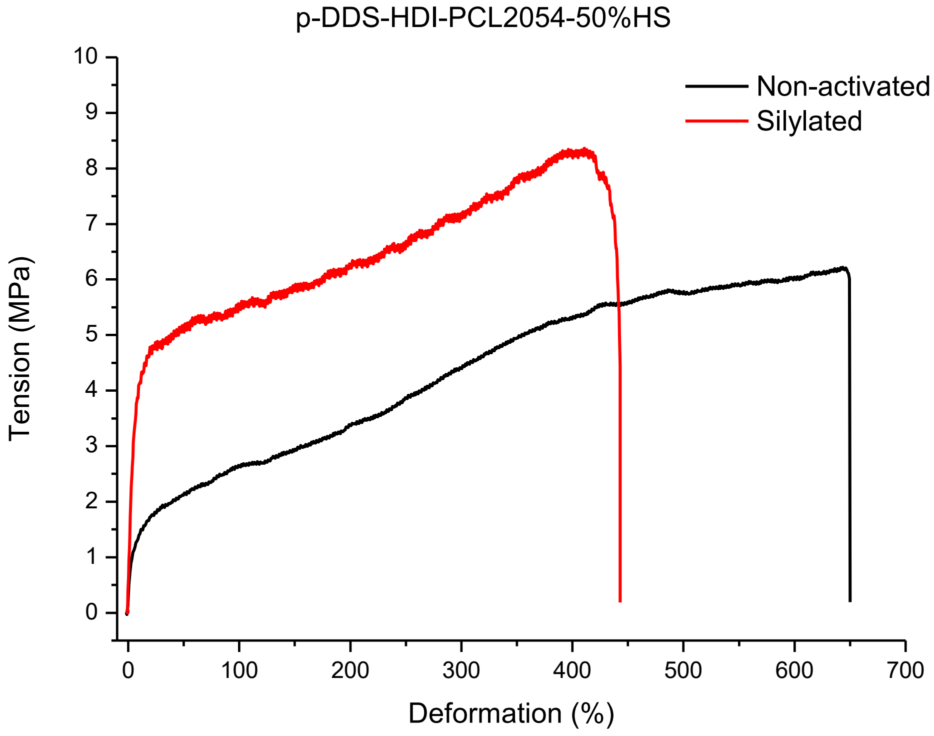

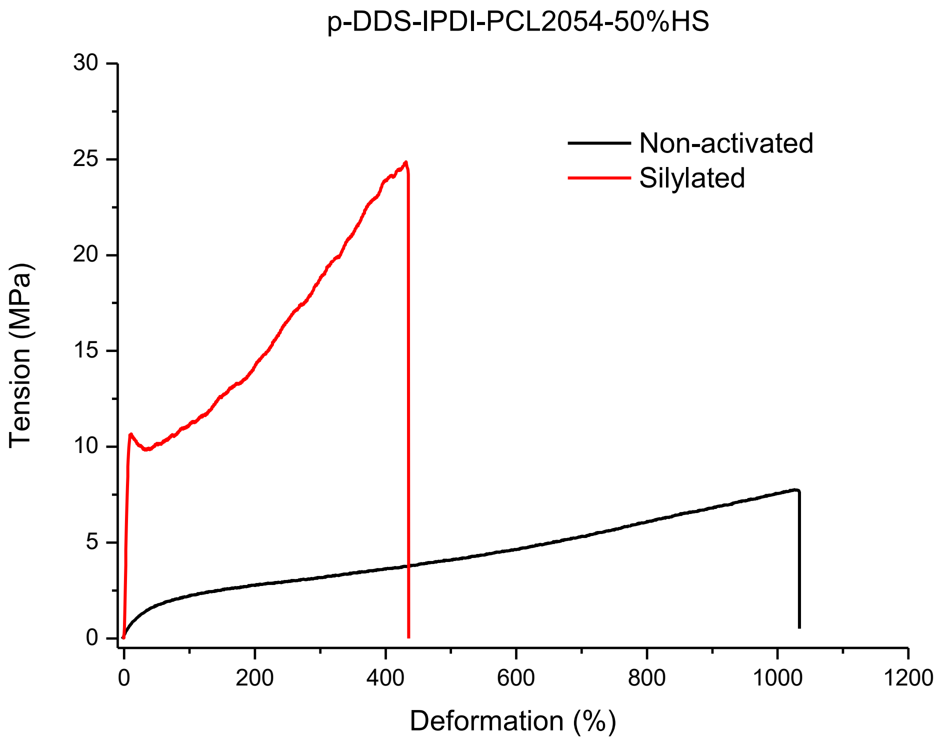

Figure SS4. The stress-strain curves of silylated and non-silylated polymers p-DDS-HDI-PCL2054-50HS and p-DDS-IPDI-PCL2054-50HS.

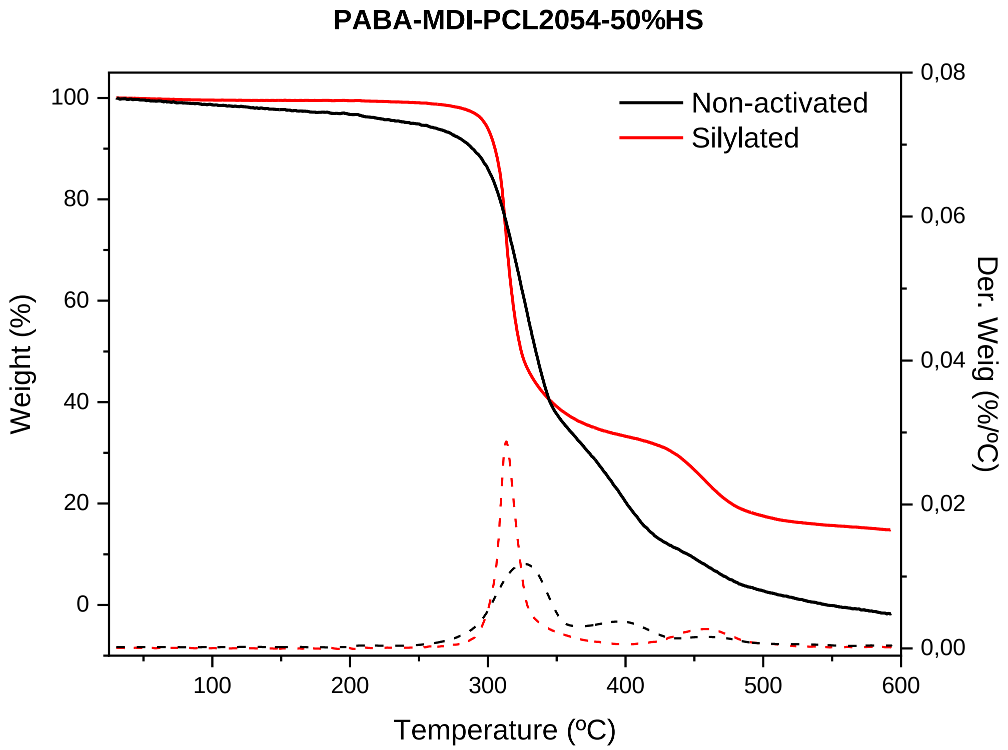

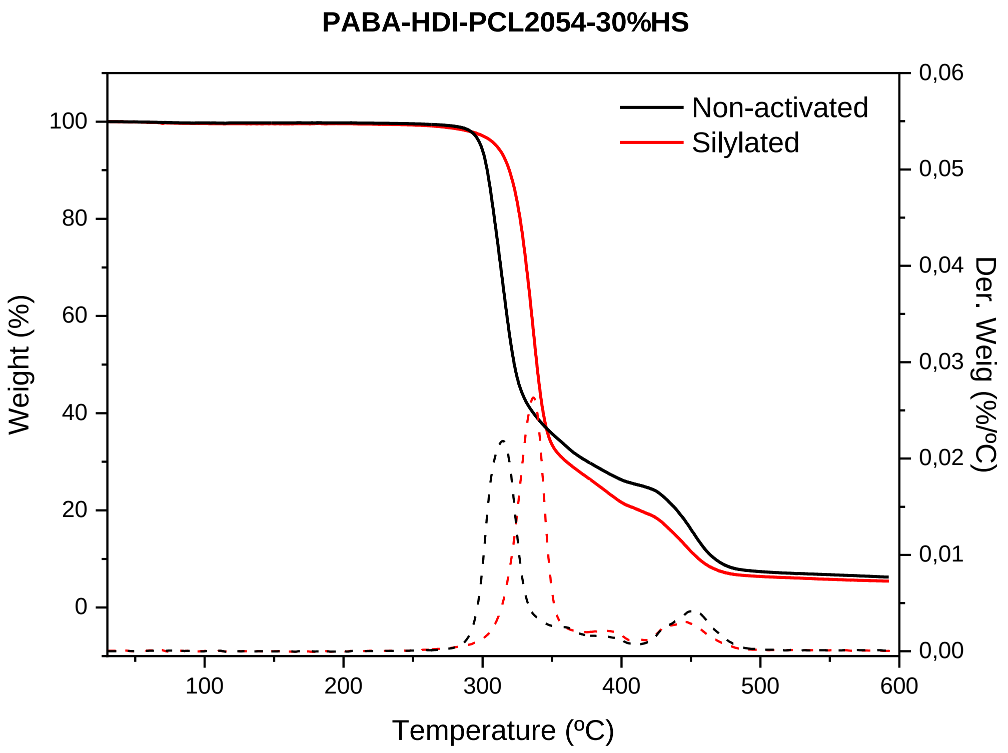

Figure S5. TGA measurements of silylated and non-silylated polymers PABA-MDI-PCL2054-50HS and PABA-HDI-PCL2054-30HS.

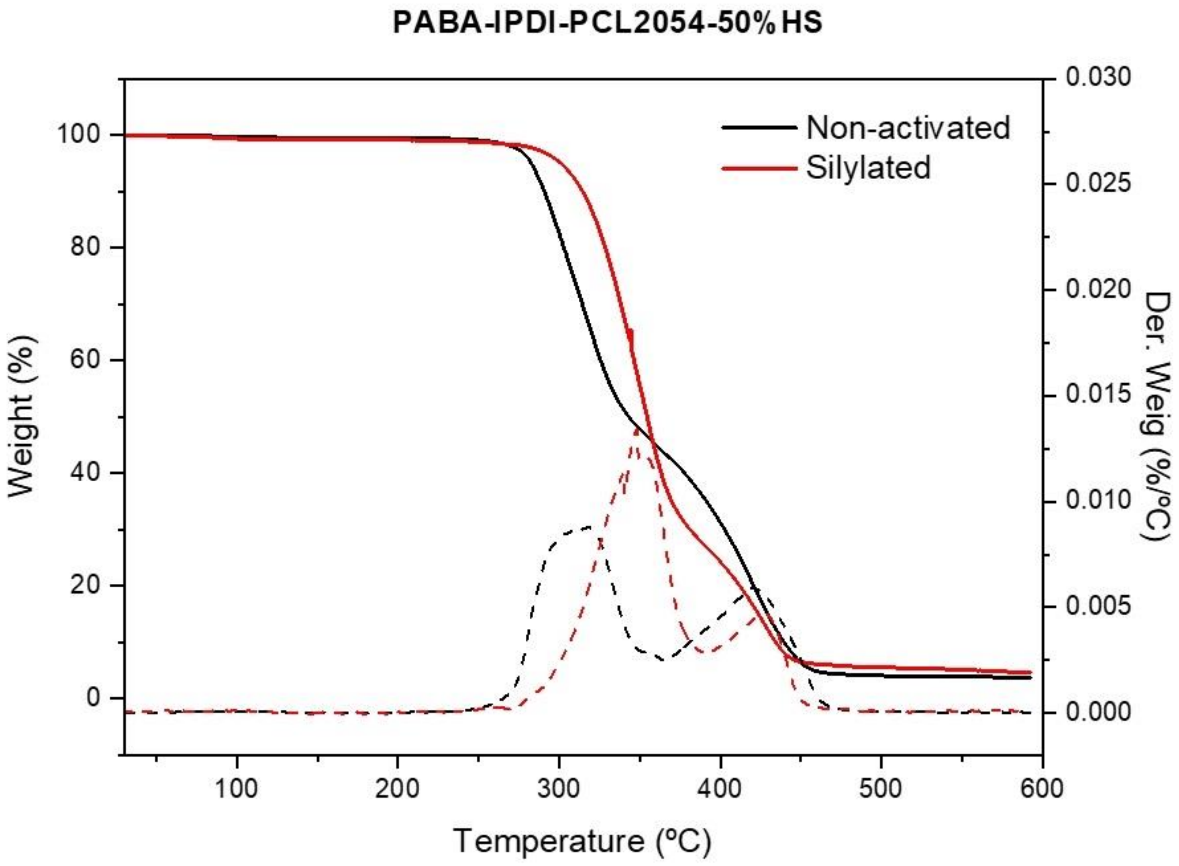

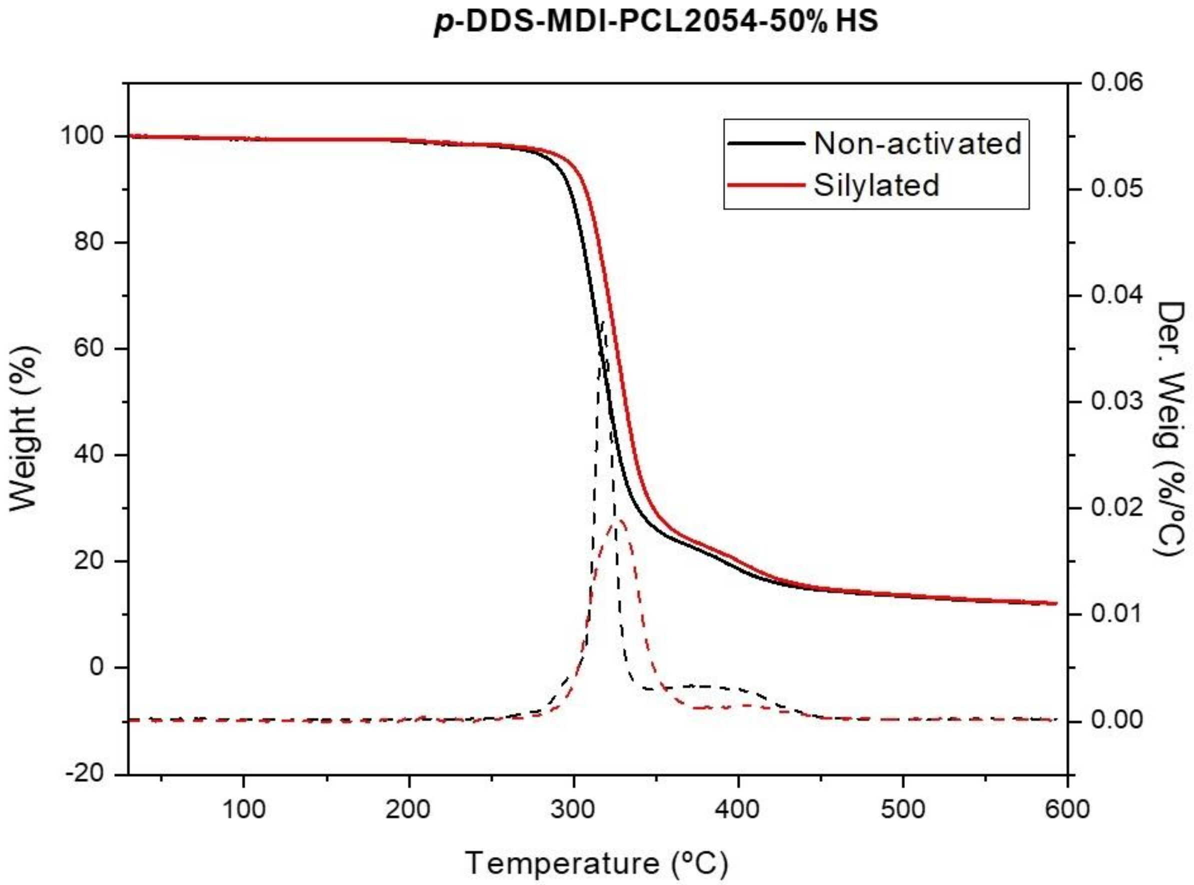

Figure S6. TGA measurements of silylated and non-silylated polymers PABA-IPDI-PCL2054-50HS and p-DDS-MDI-PCL2054-50HS.

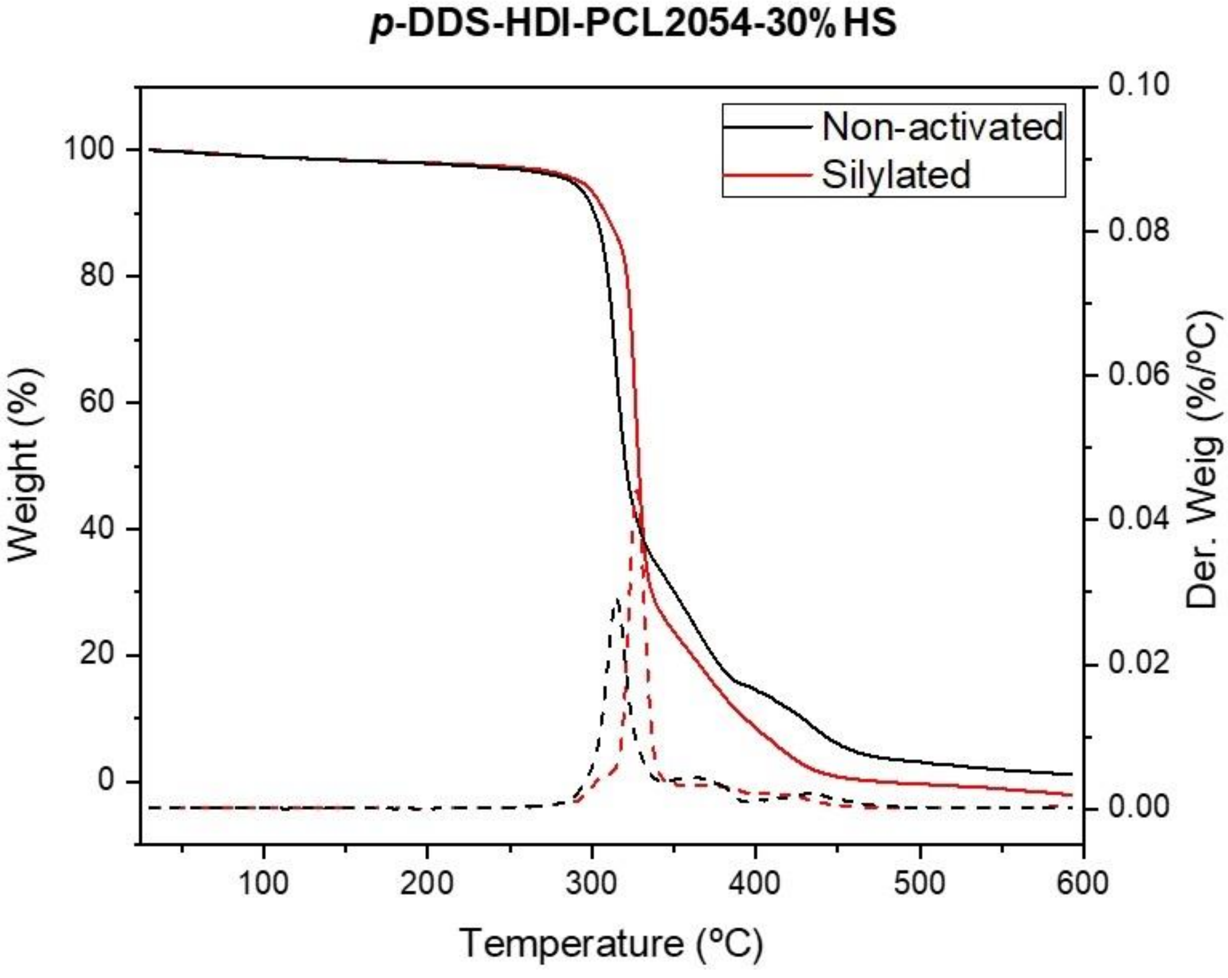

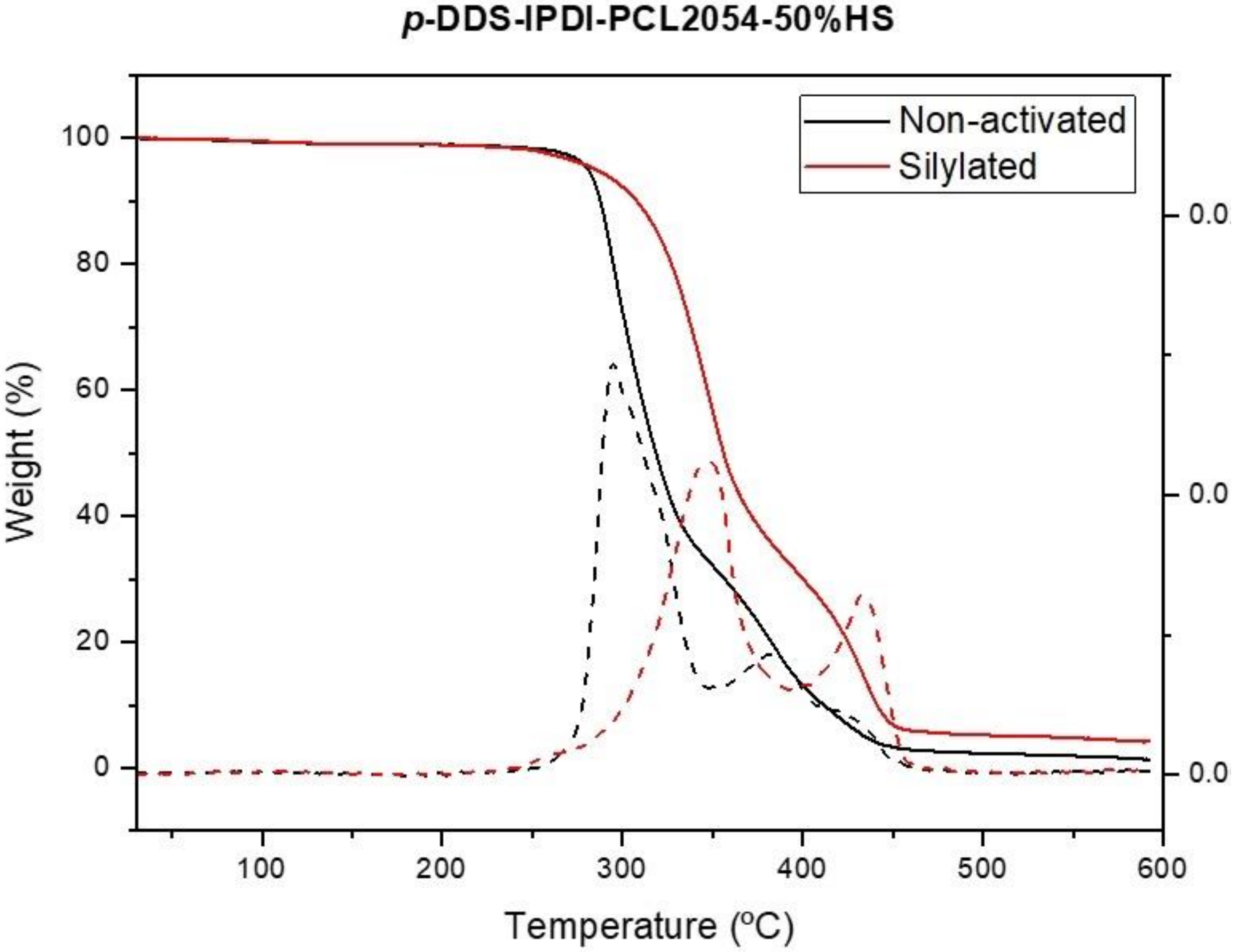

Figure S7. TGA measurements of silylated and non-silylated polymers p-DDS-HDI-PCL2054-30HS and p-DDS-IPDI-PCL2054-50HS.

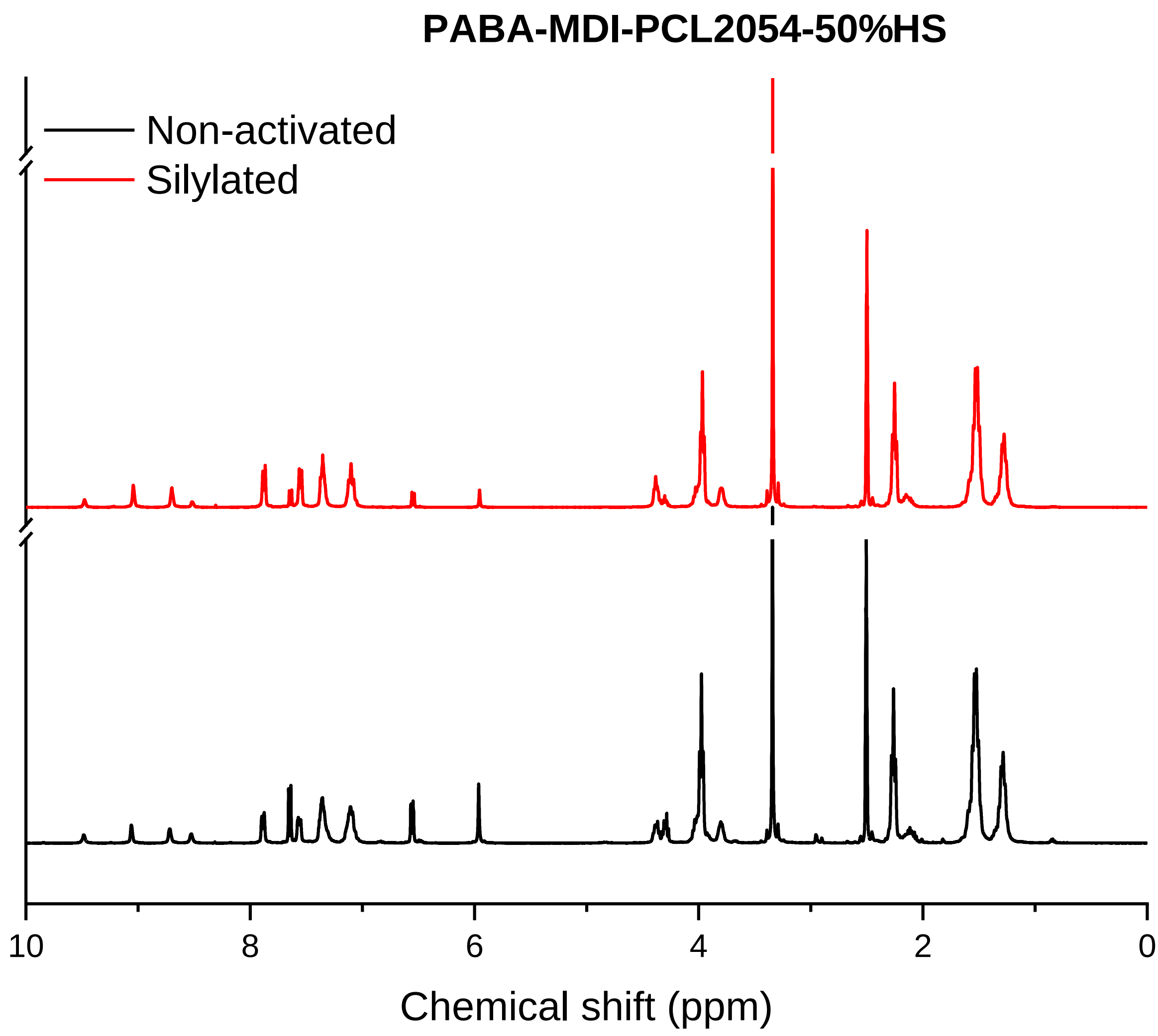

Figure S8. Comparison of ^{1}H-NMR spectra of polymer PABA-MDI-PCL2054-50HS by silylation and standard protocol.

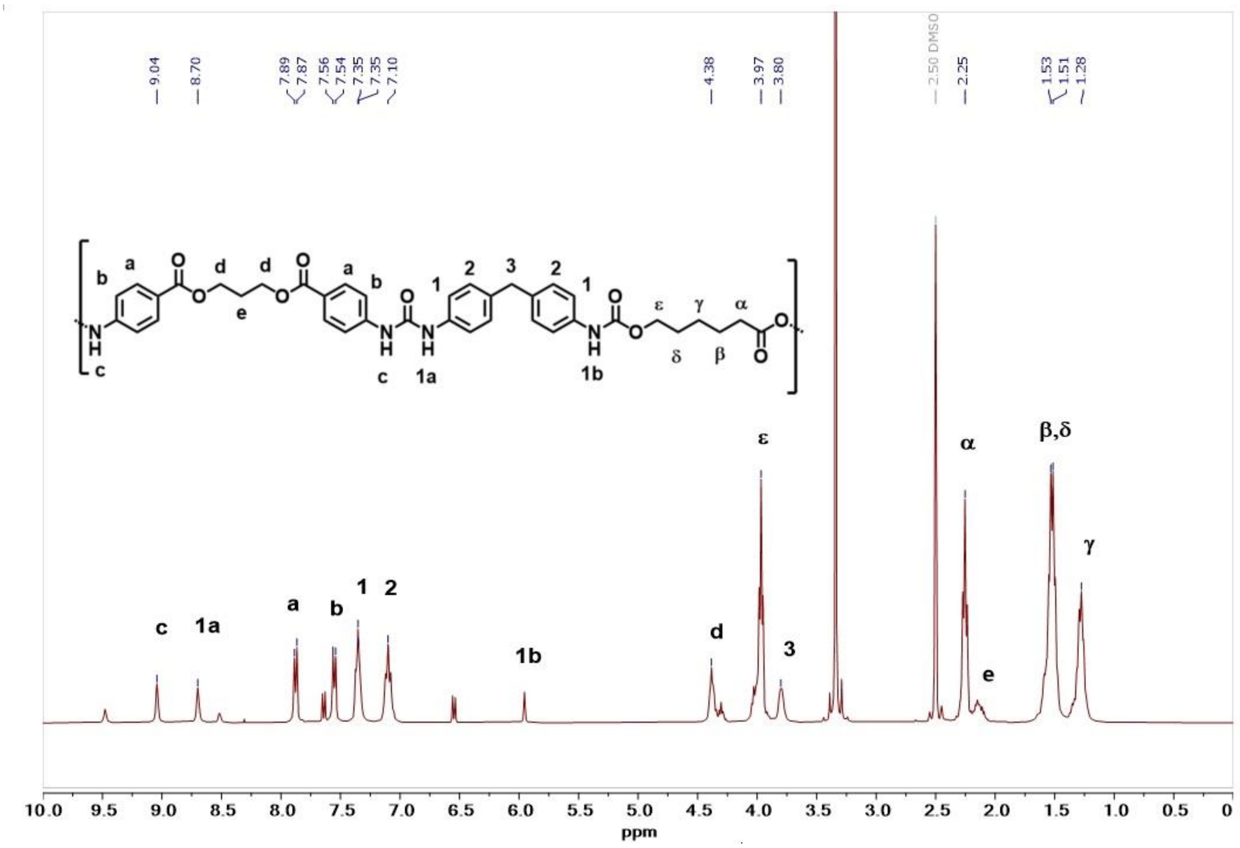

Figure S9. ^{1}H-NMR spectrum of polyurethane PABA-MDI-PCL2054-50HS.

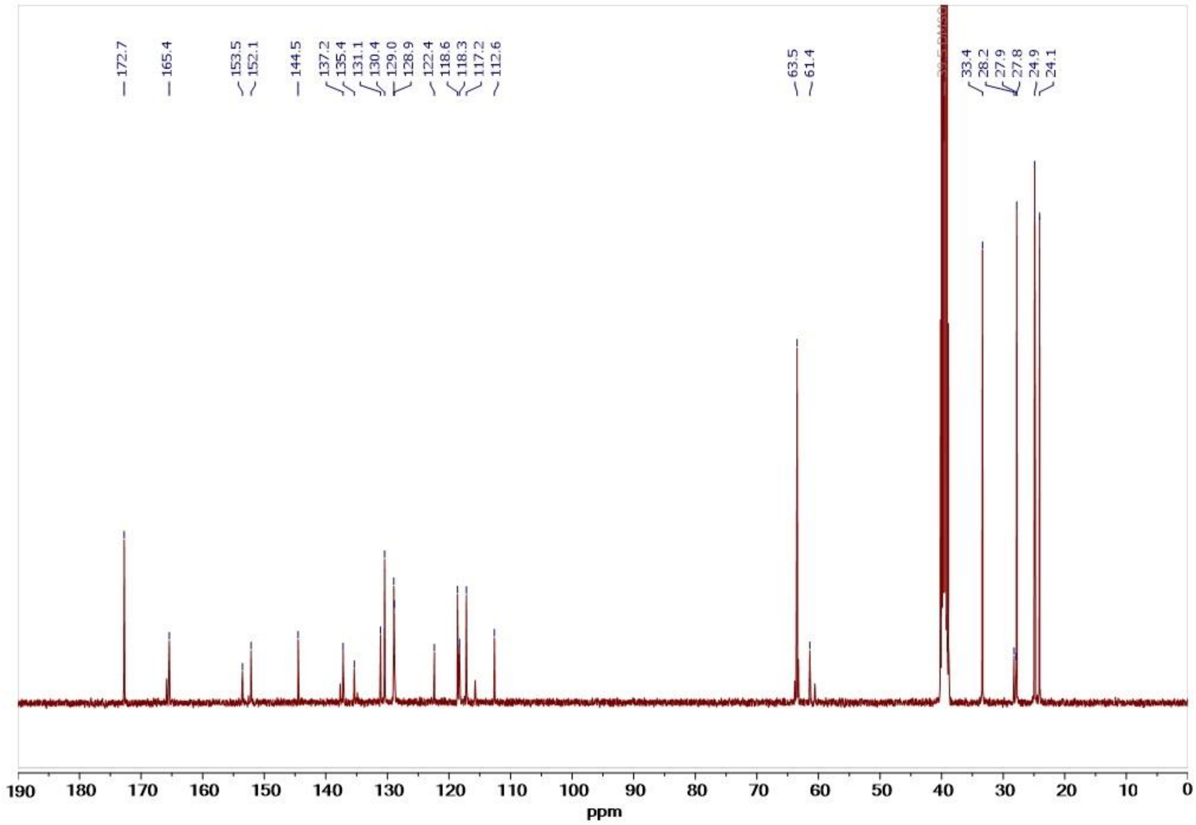

Figure S10. ^{13}C-NMR spectrum of polyurethane PABA-MDI-PCL2054-50HS.

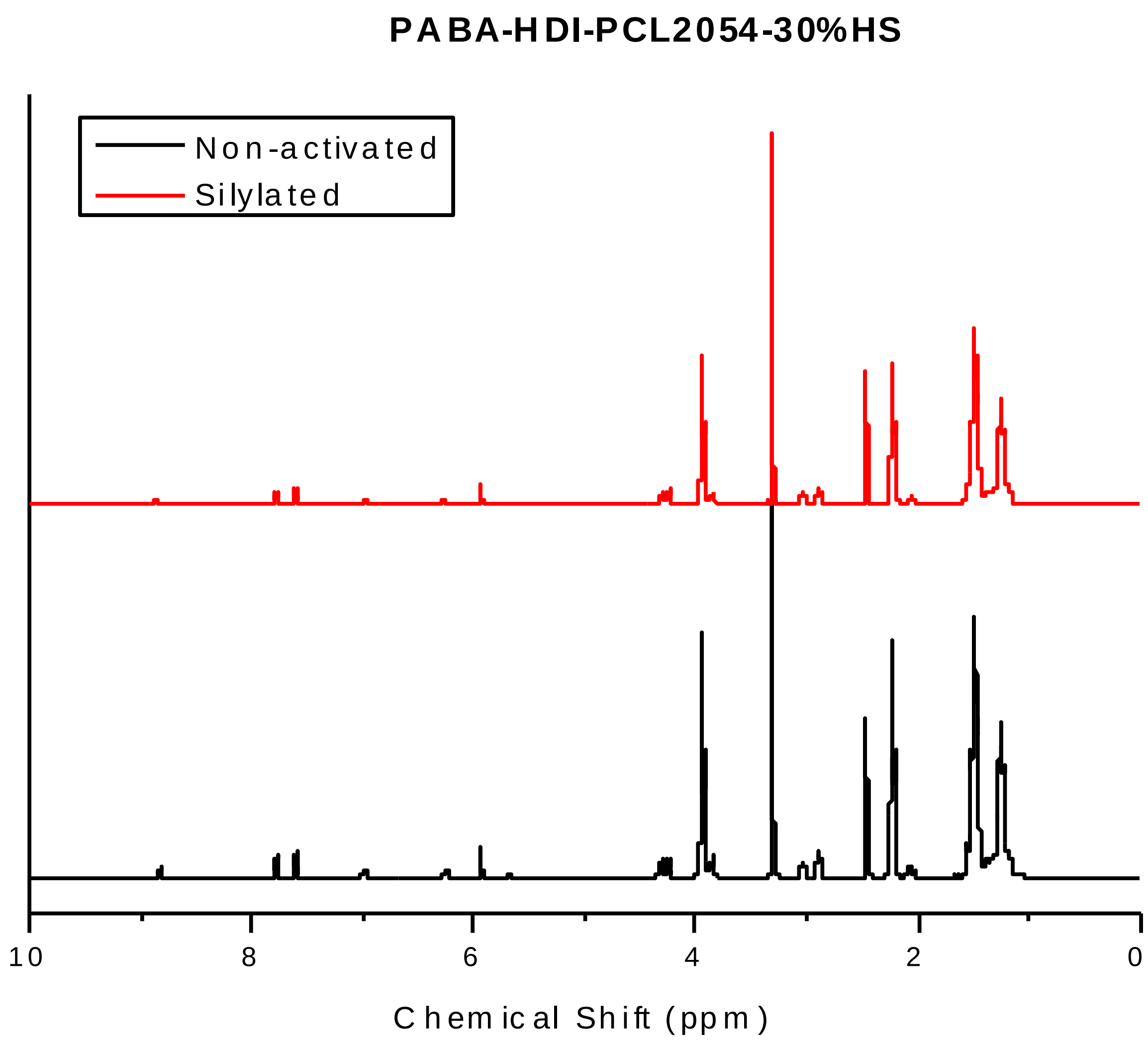

Figure S11. Comparison of ^{1}H-NMR spectra of polymer PABA-HDI-PCL2054-30HS by silylation and standard protocol.

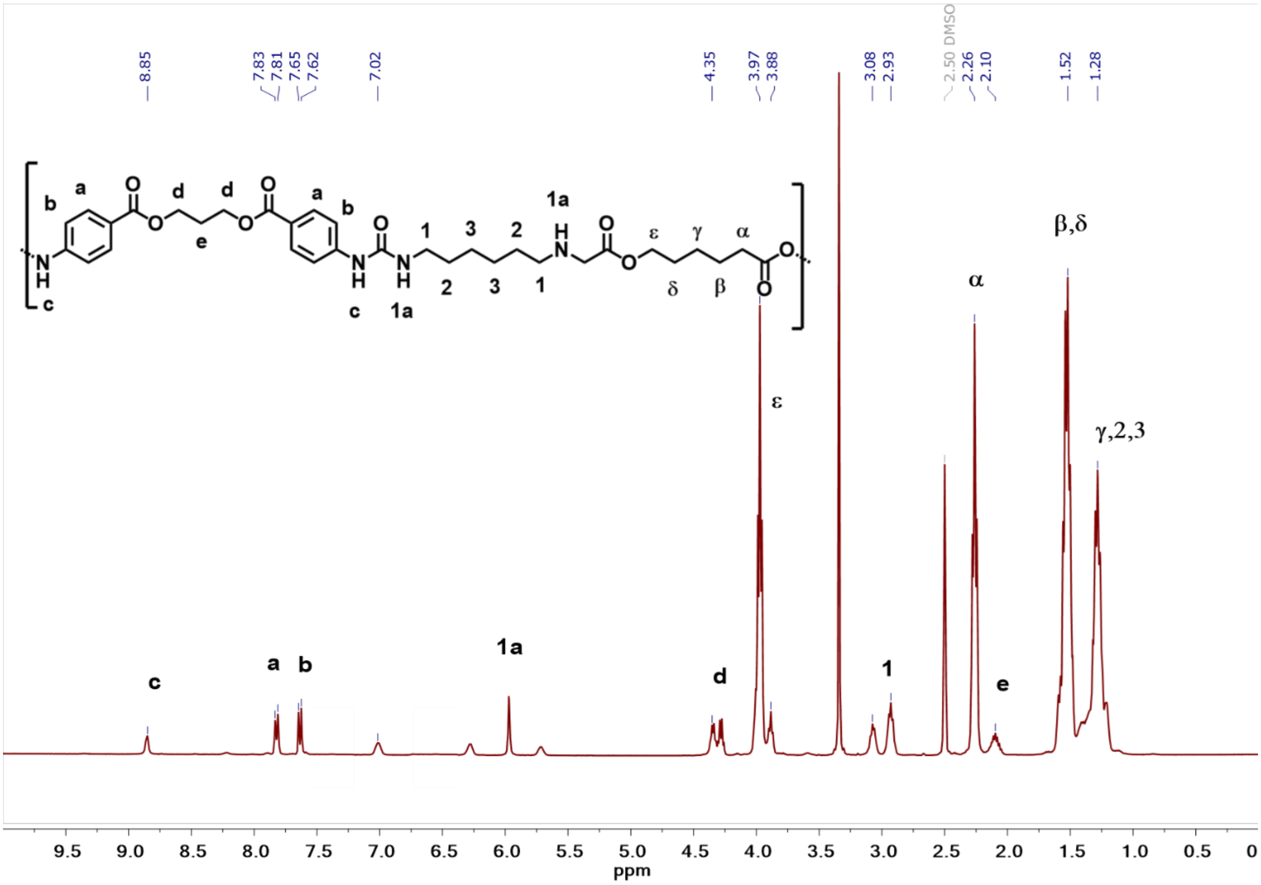

Figure S12. ^{1}H-NMR spectrum of polyurethane PABA-HDI-PCL2054-30HS.

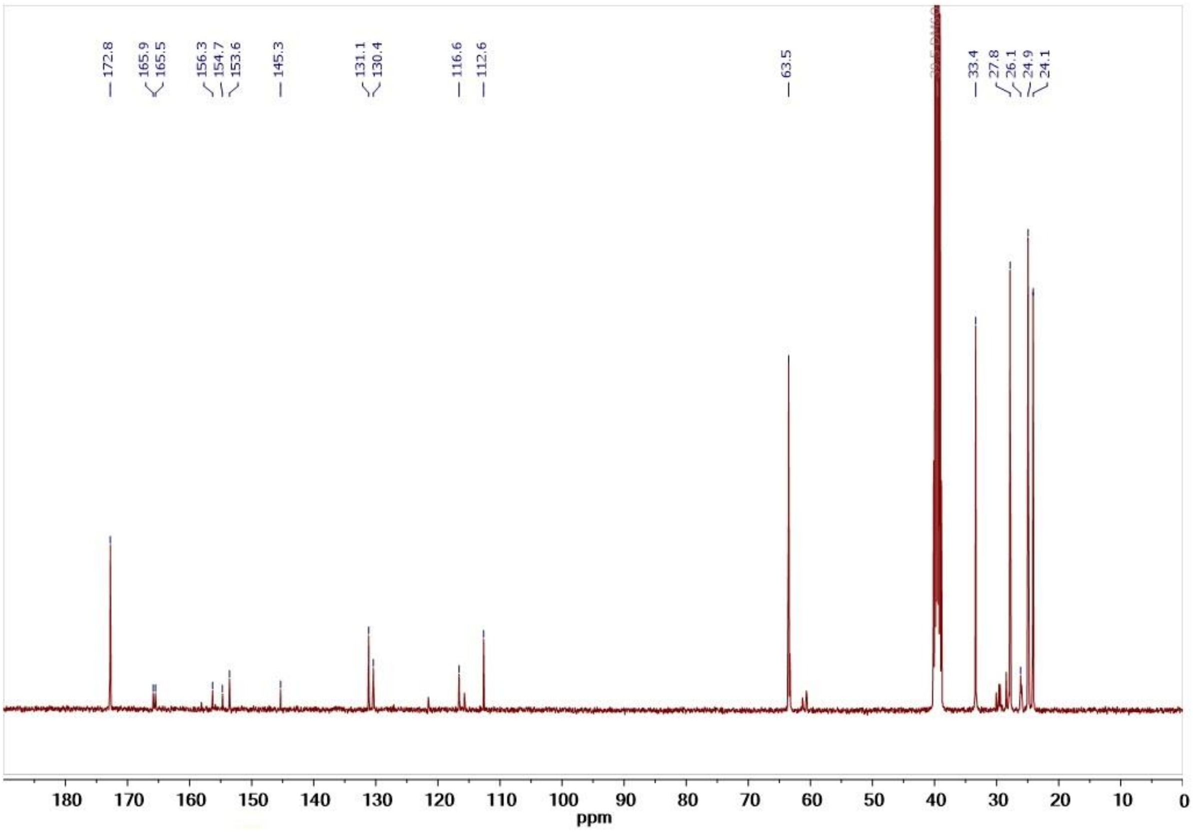

Figure S13. ^{13}C-NMR spectrum of polyurethane PABA-HDI-PCL2054-30HS.

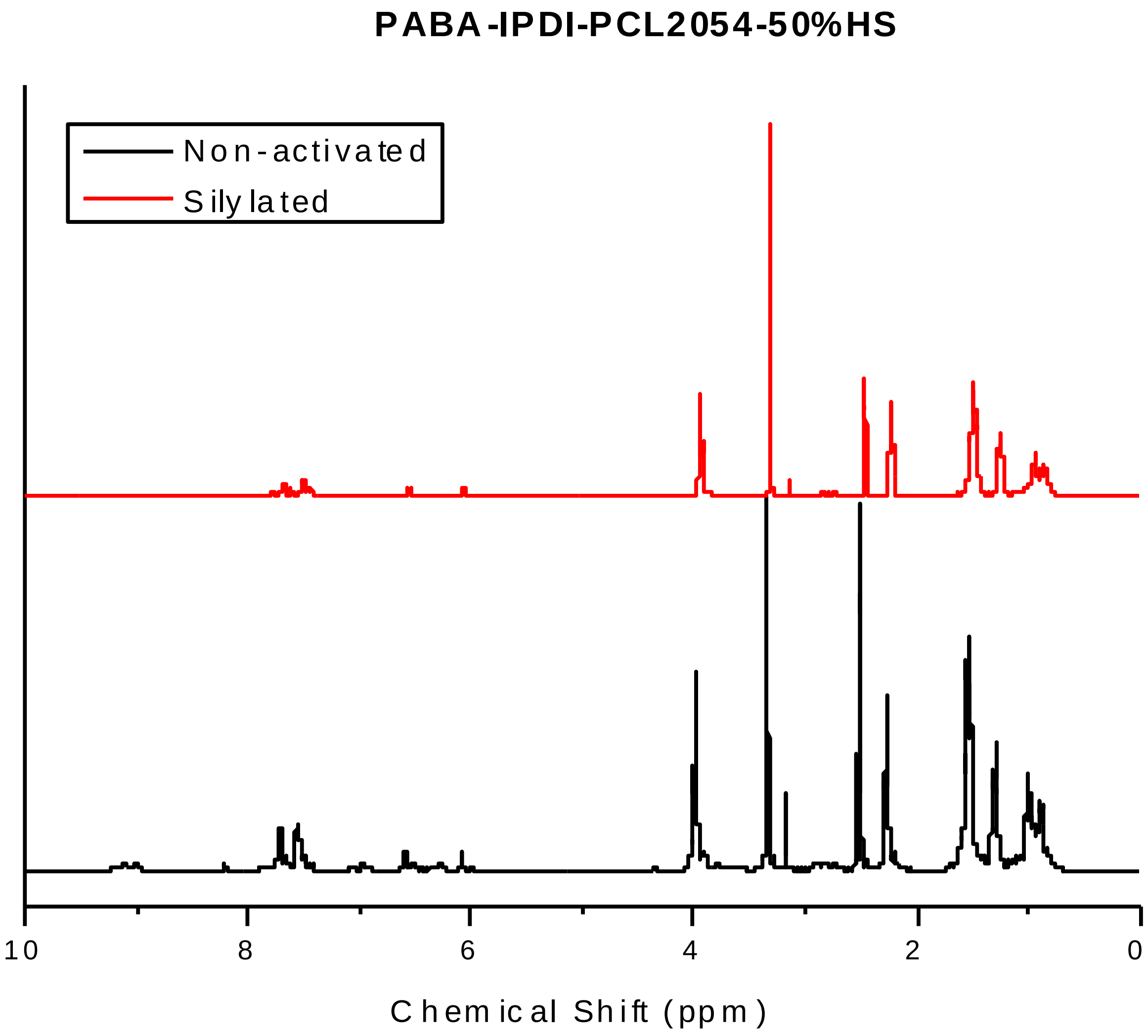

Figure S14. Comparison of ^{1}H-NMR spectra of polymer PABA-IPDI-PCL2054-50HS by silylation and standard protocols.

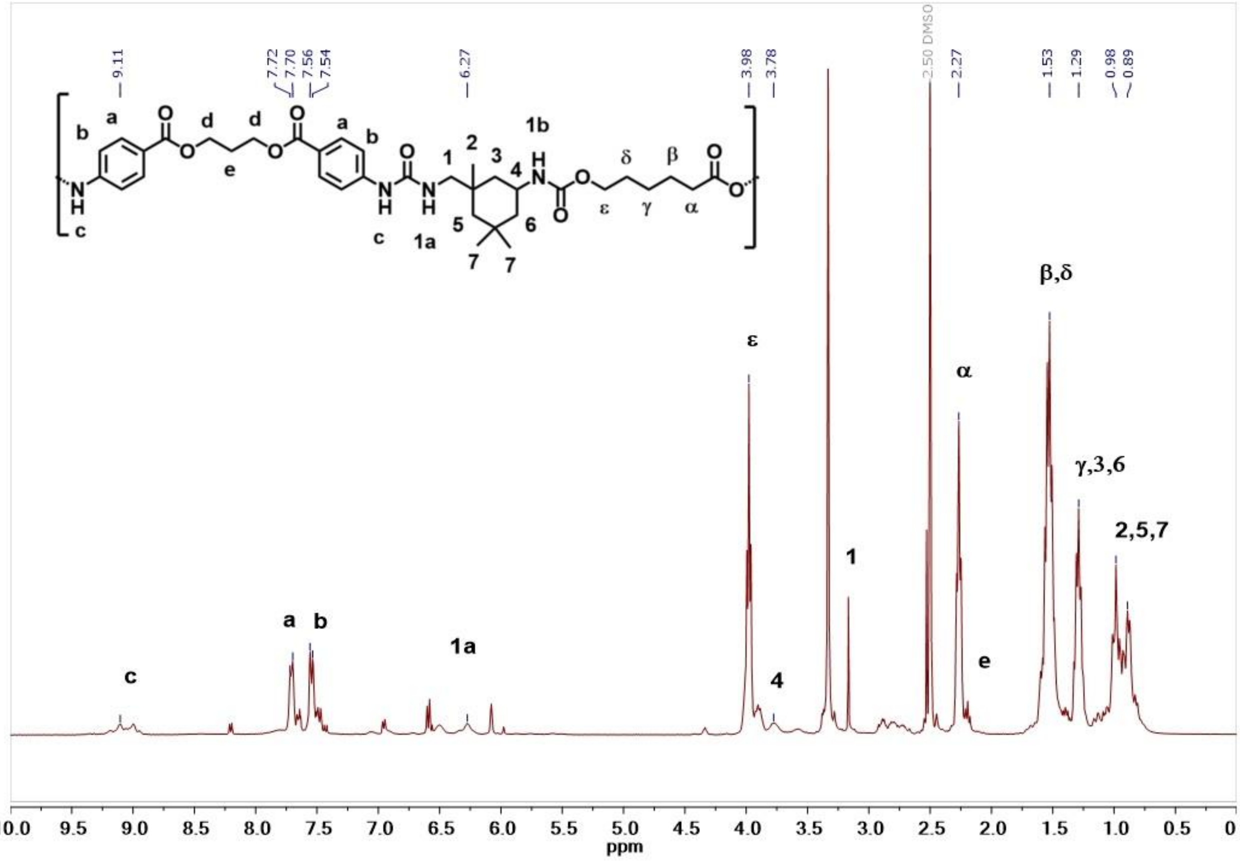

Figure S15. ^{1}H-NMR spectrum of polyurethane PABA-IPDI-PCL2054-50HS.

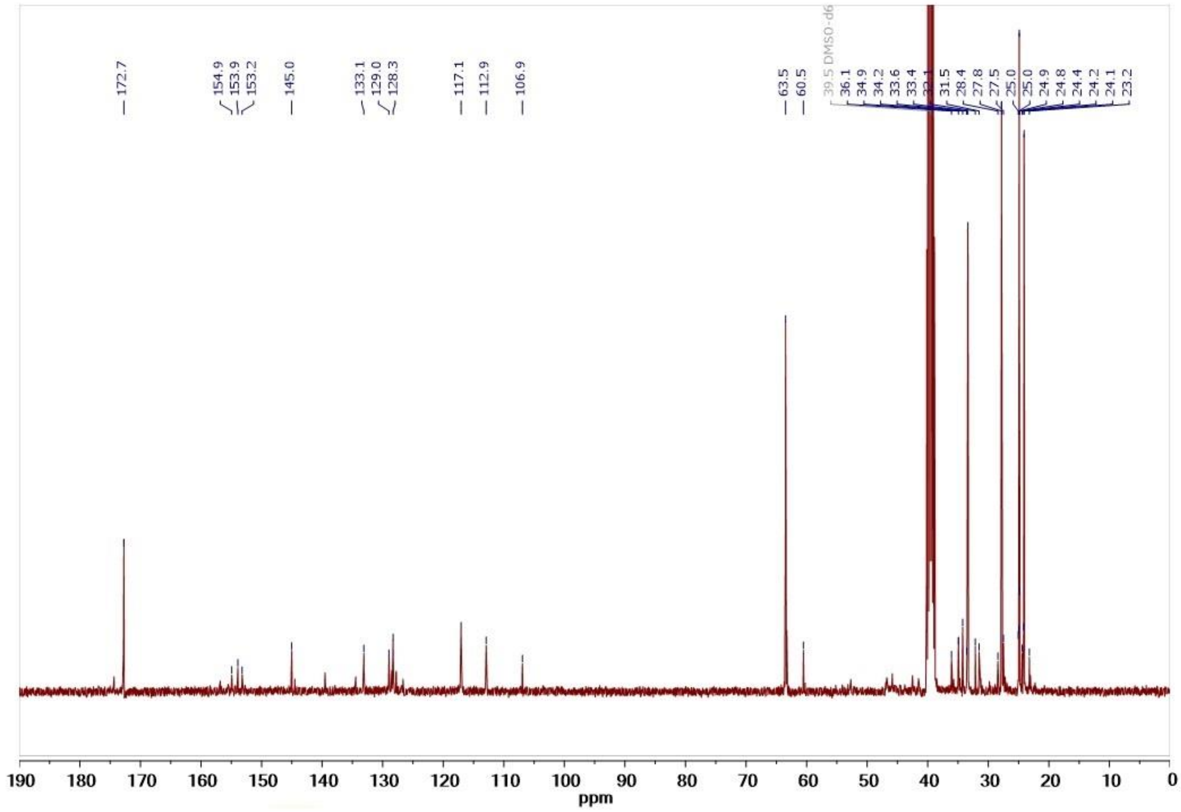

Figure S16. ^{13}C-NMR spectrum of polyurethane PABA-IPDI-PCL2054-50HS.

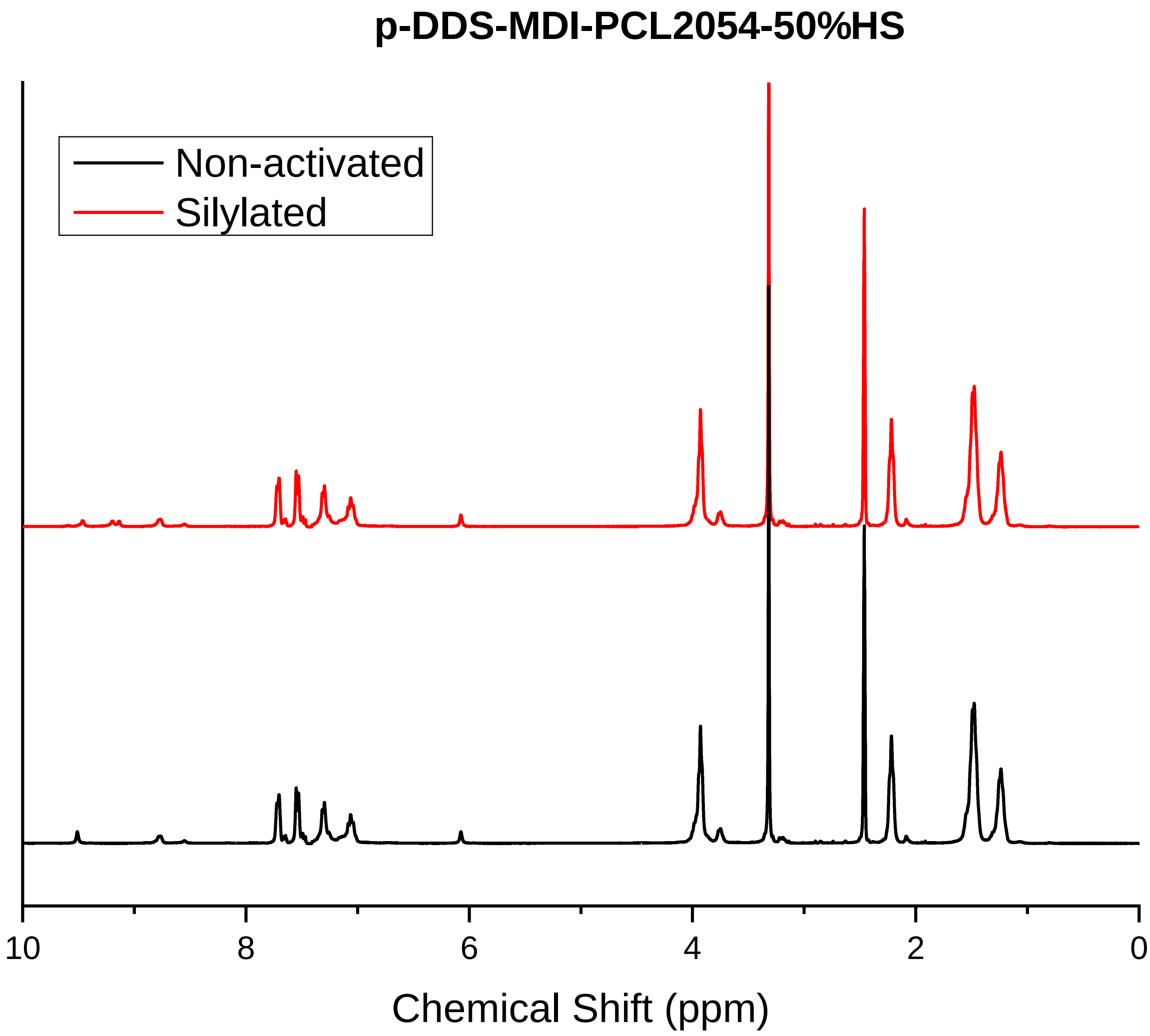

Figure S17. Comparison of 1H-NMR spectra of polymer p-DDS-MDI-PCL2054-50HS by silylation and standard protocols.

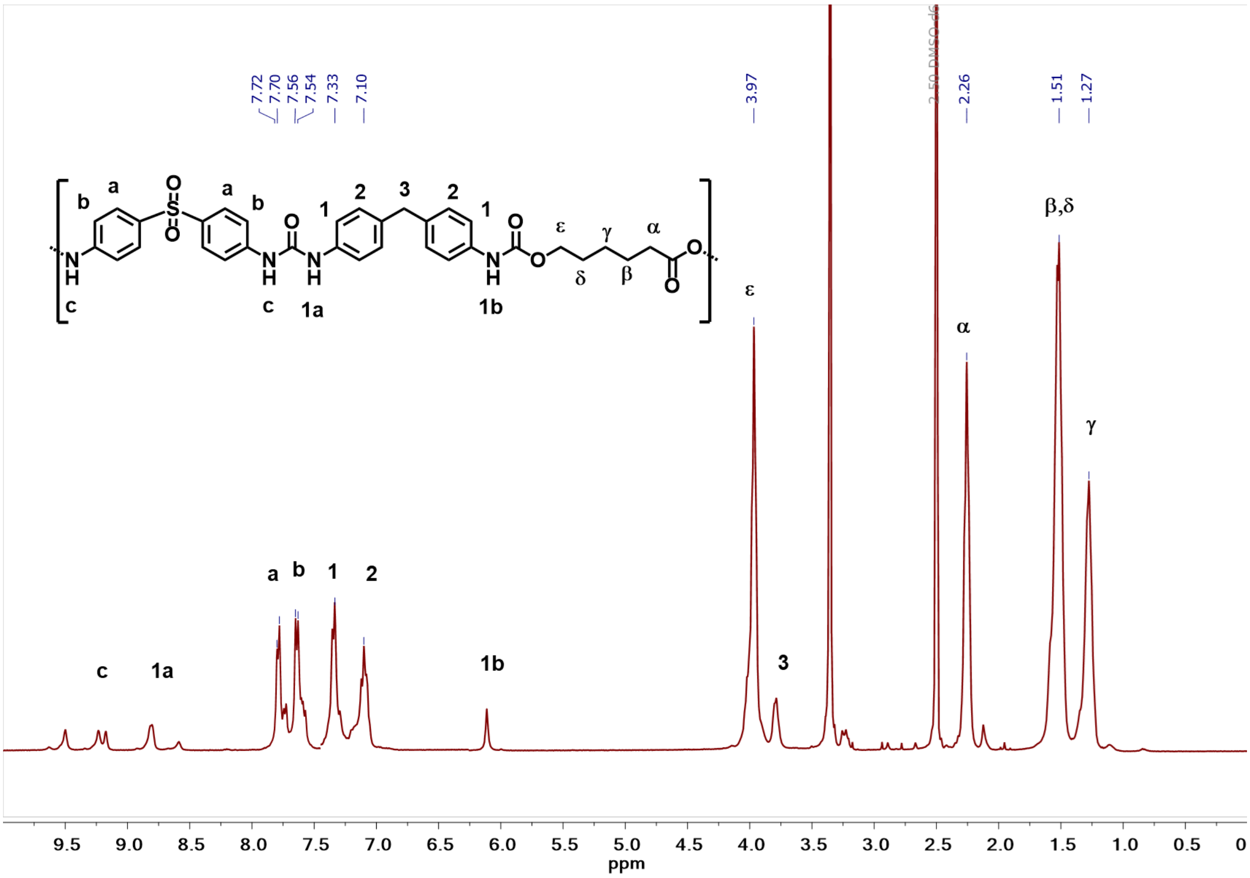

Figure S18. ^{1}H-NMR spectrum of polyurethane p-DDS-MDI-PCL2054-50HS.

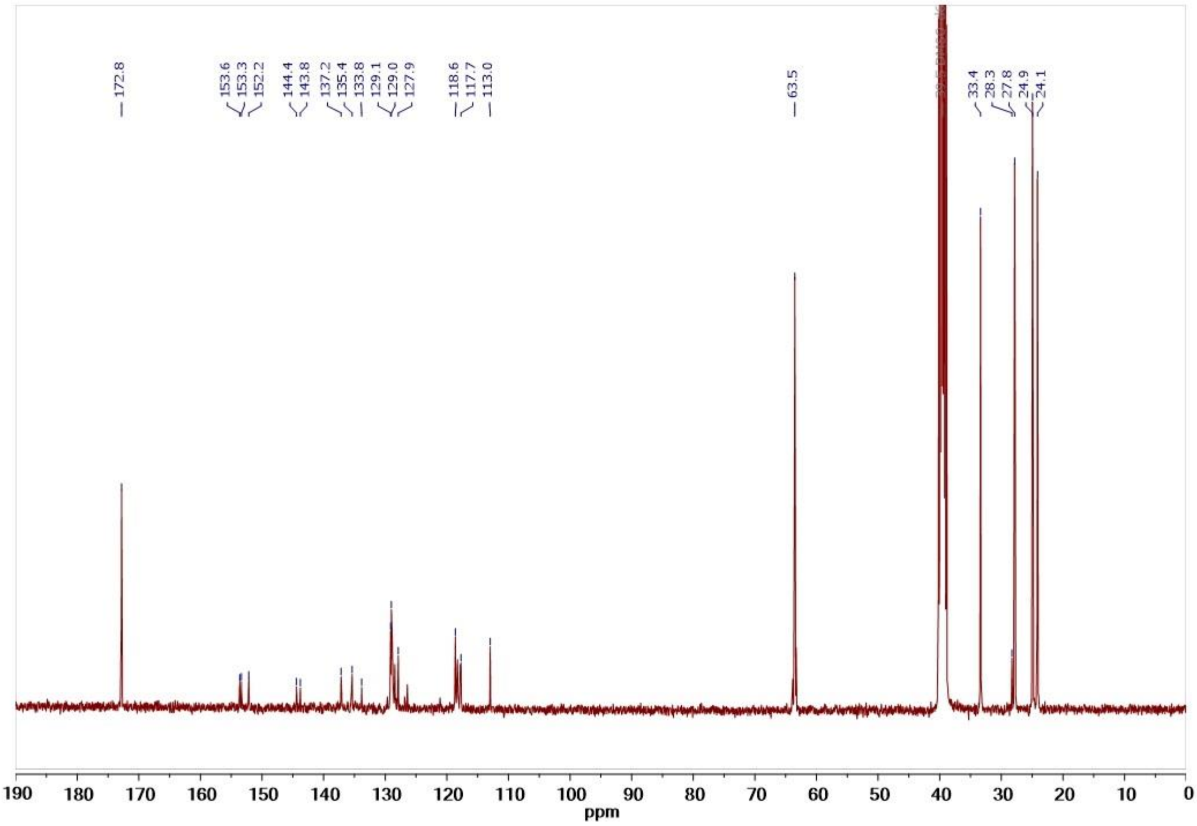

Figure S19. ^{13}C-NMR spectrum of polyurethane p-DDS-MDI-PCL2054-50HS.

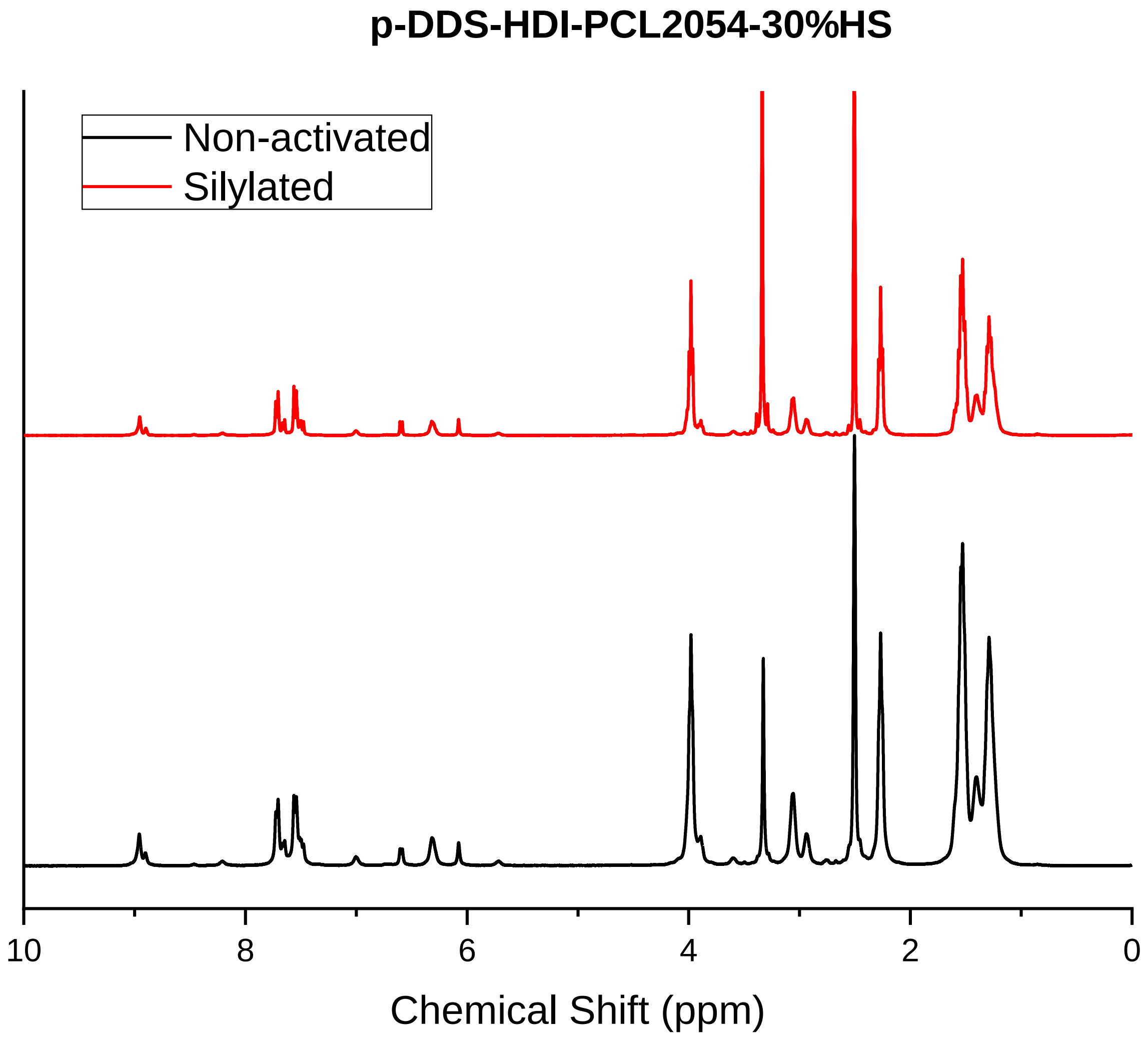

Figure S20. Comparison of ^{1}H-NMR spectra of polymer p-DDS-HDI-PCL2054-30HS by silylation and standard protocols.

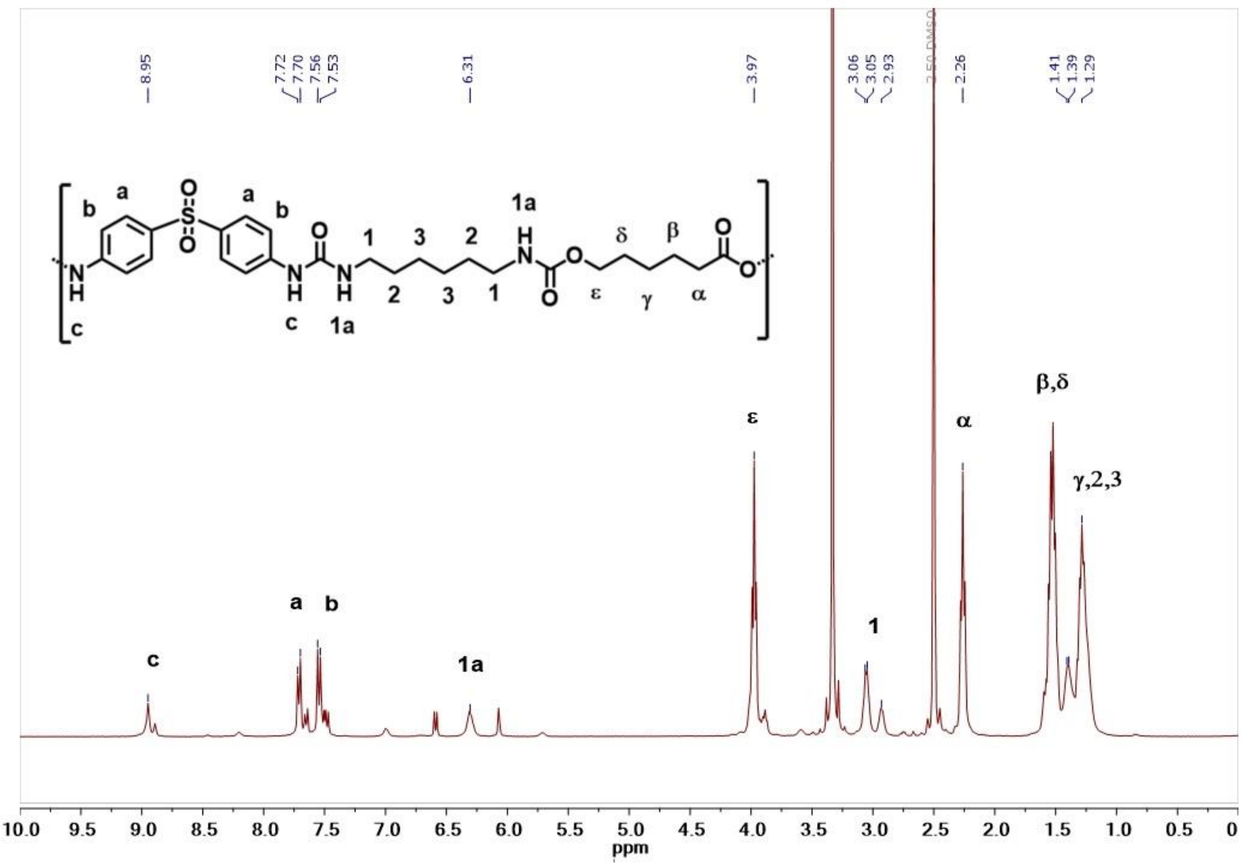

Figure S21. ^{1}H-NMR spectrum of polyurethane p-DDS-HDI-PCL2054-30HS.

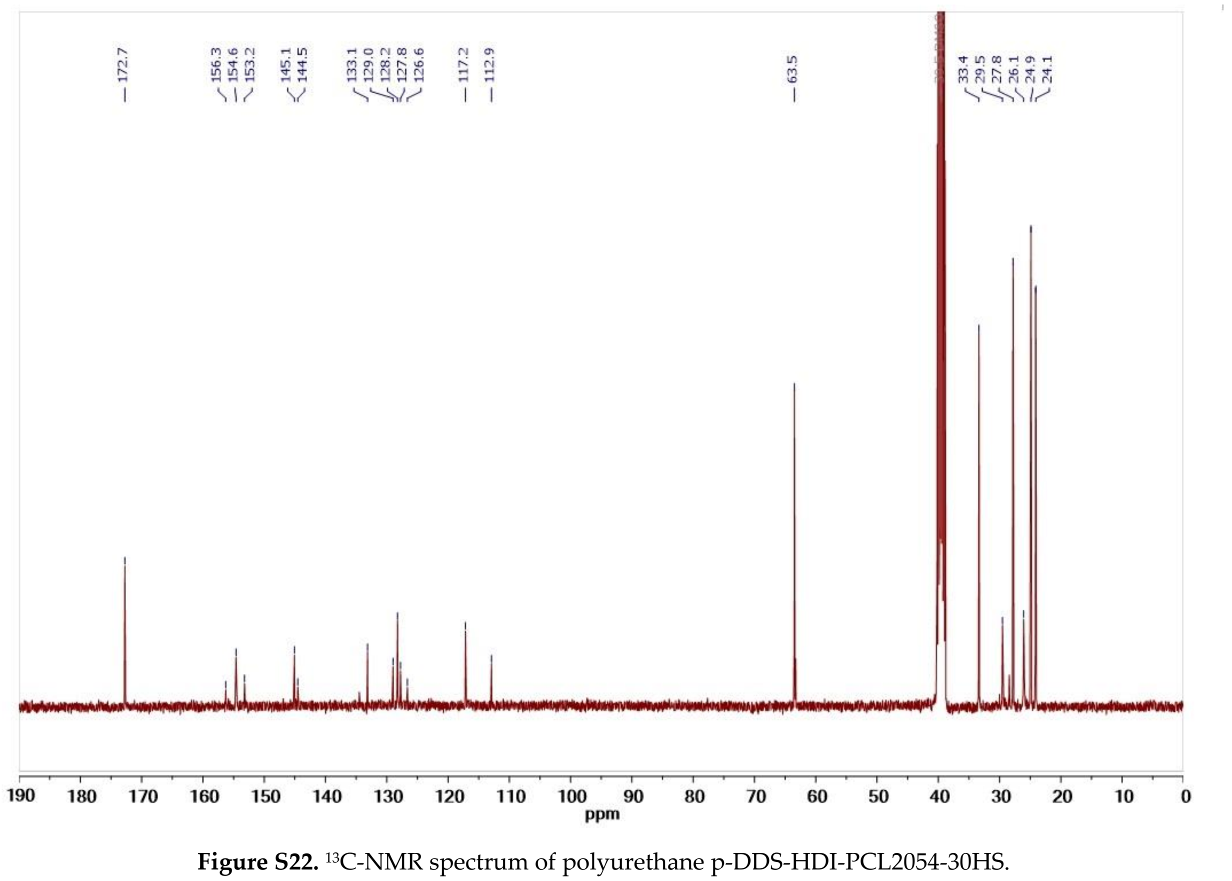

Figure S22. ^{13}C-NMR spectrum of polyurethane p-DDS-HDI-PCL2054-30HS.

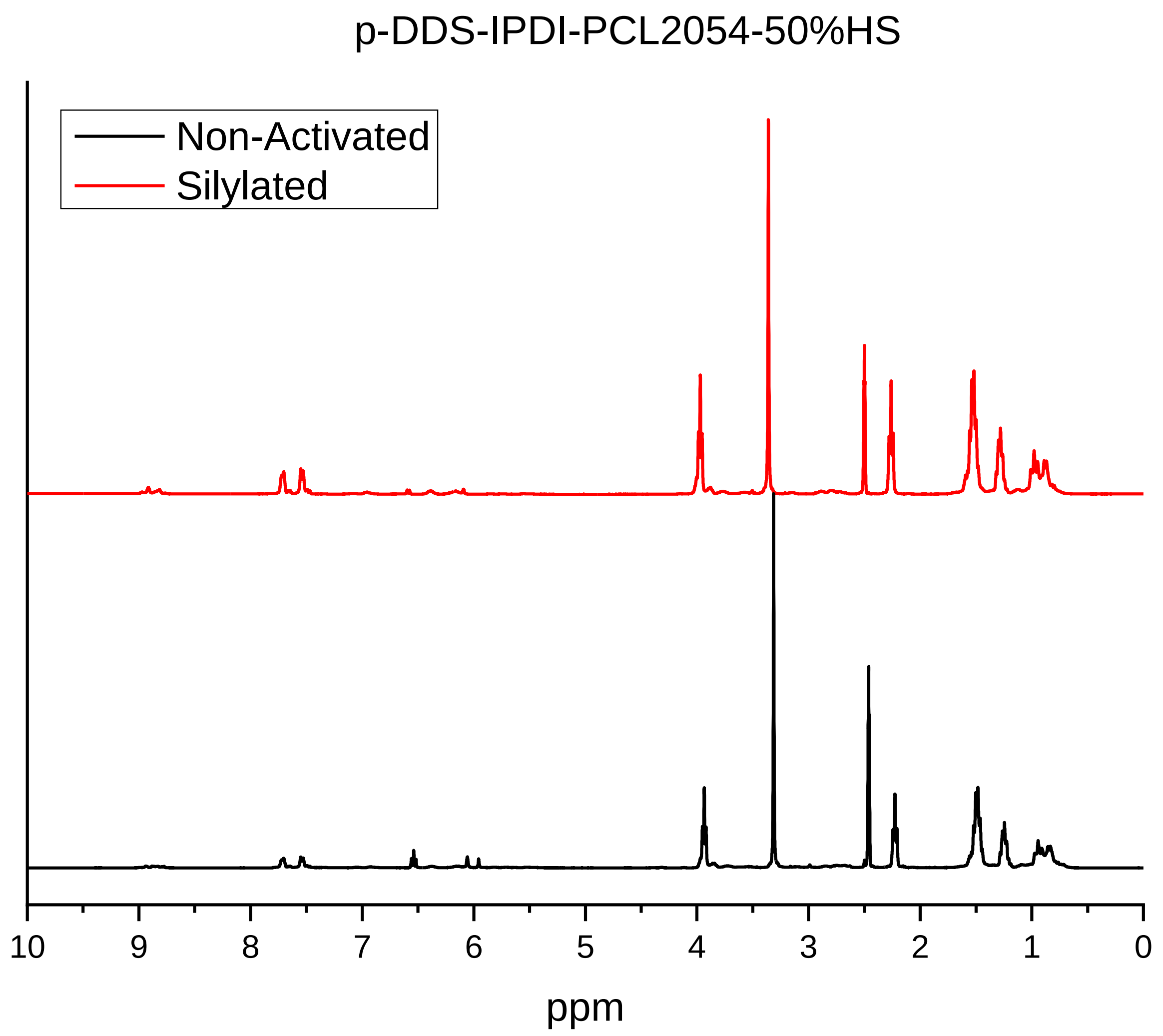

Figure S23. Comparison of ^{1}H-NMR spectra of polymer p-DDS-IPDI-PCL2054-50HS by silylation and standard protocols.

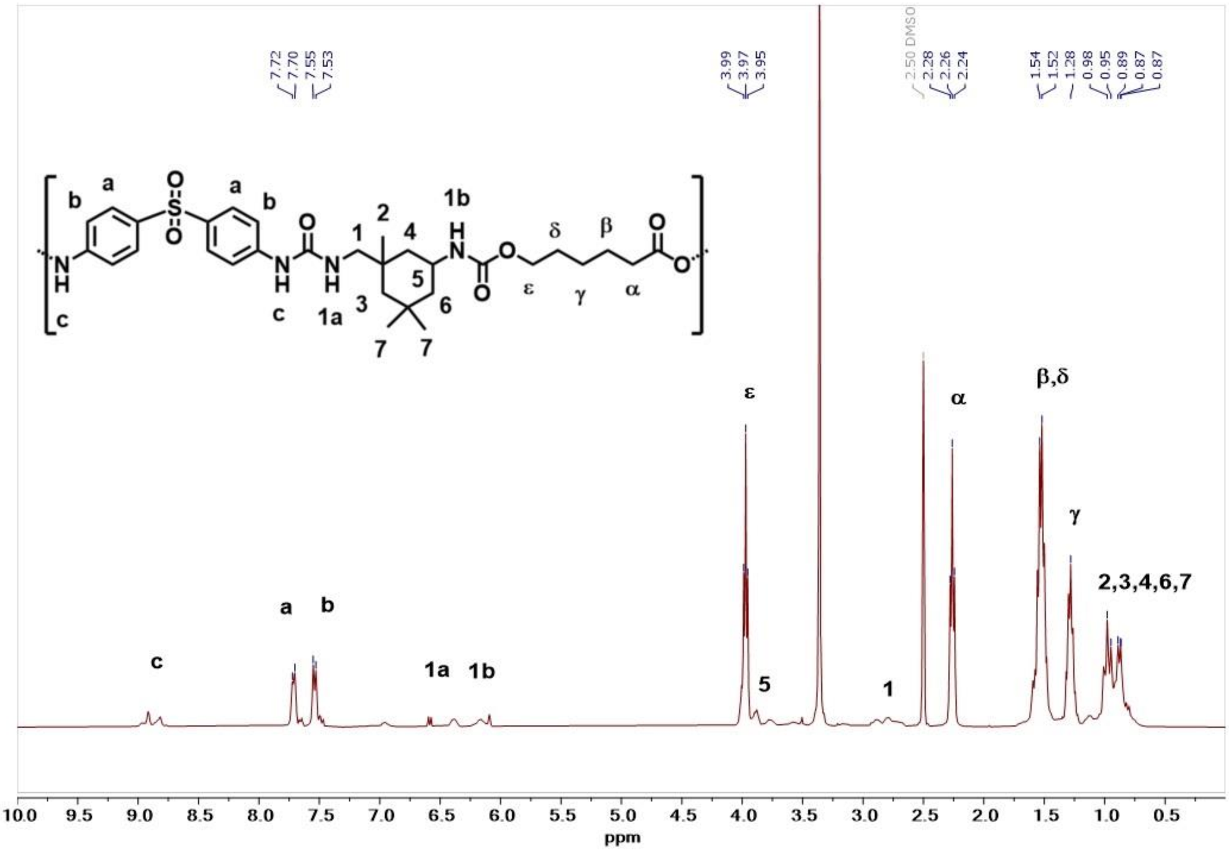

Figure S24. ^{1}H-NMR spectrum of polyurethane p-DDS-IPDI-PCL2054-50HS.

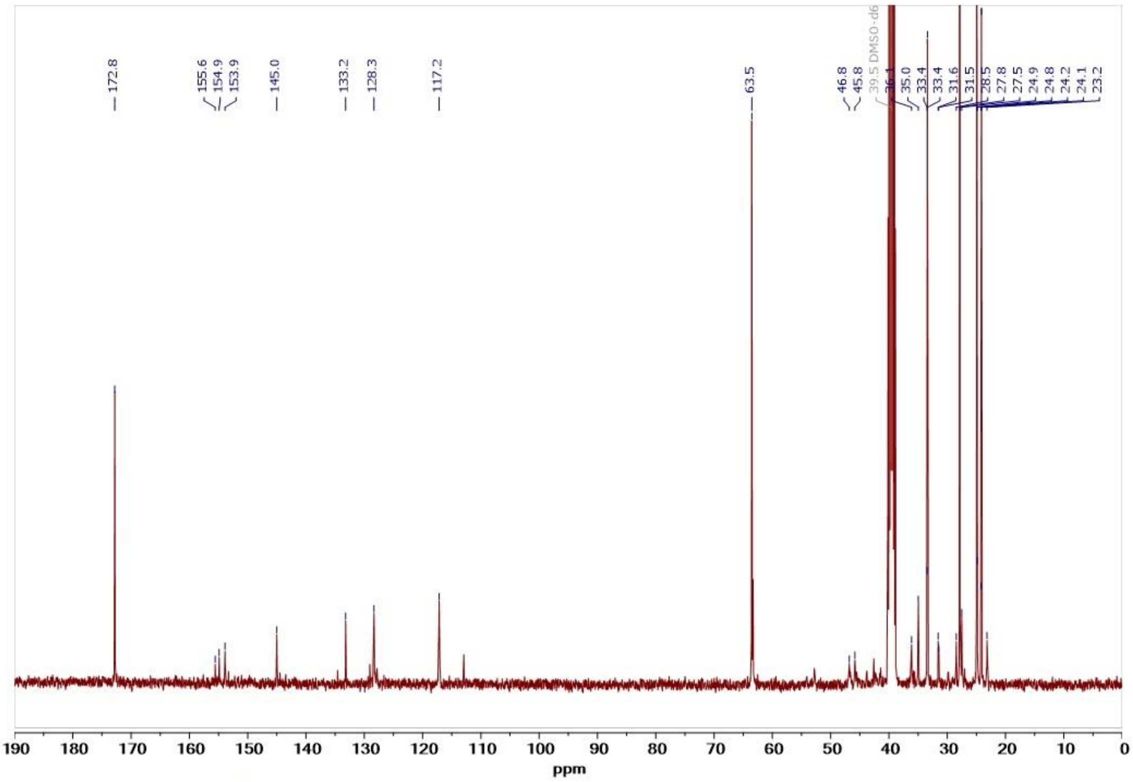

Figure S25. ^{13}C-NMR spectrum of polyurethane p-DDS-IPDI-PCL2054-50HS.

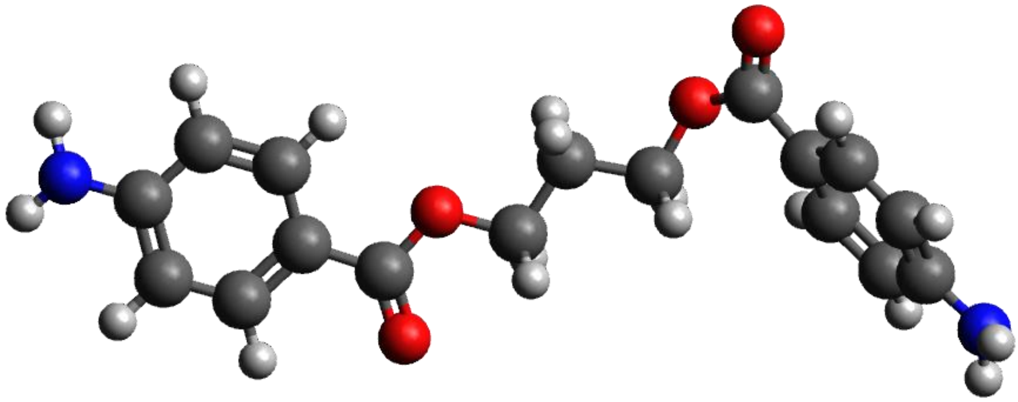

Figure S26. Chemical structure of pristine diamine PABA.

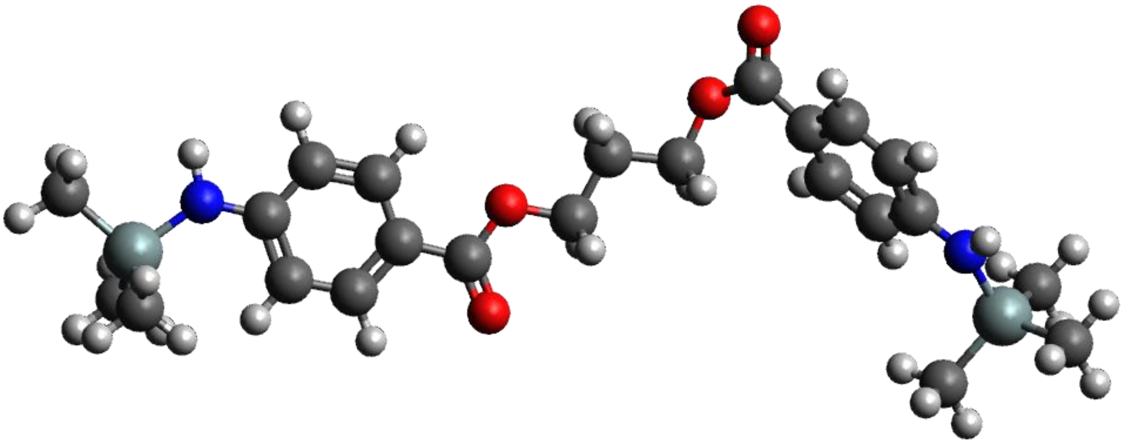

Figure S27. Chemical structure of silylated diamine PABA.

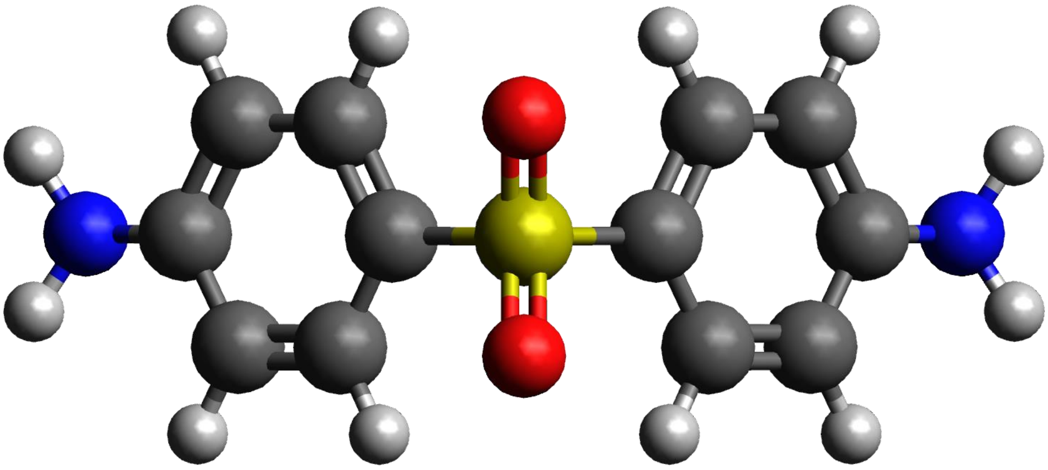

Figure S28. Chemical structure of pristine diamine p-DDS.

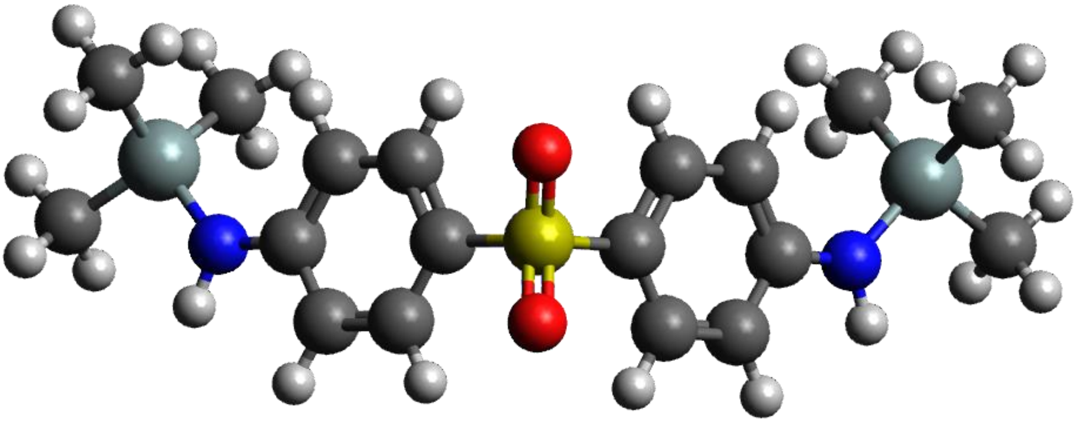

Figure S29. Chemical structure of silylated diamine p-DDS.

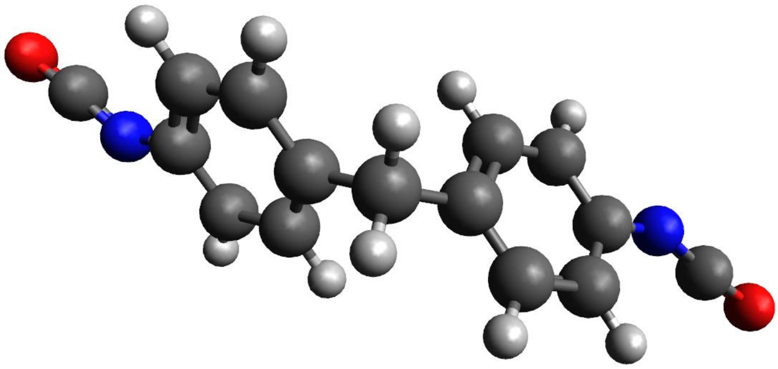

Figure S30. Chemical structure of aromatic diisocyanate MDI.

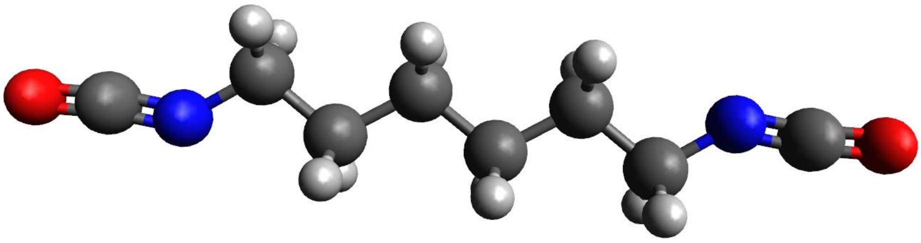

Figure S31. Chemical structure of aliphatic diisocyanate HDI.

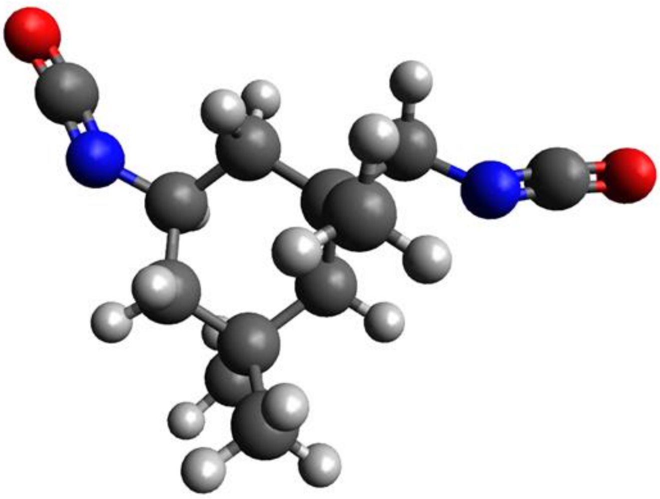

Figure S32. Chemical structure of aliphatic hindered diisocyanate IPDI.

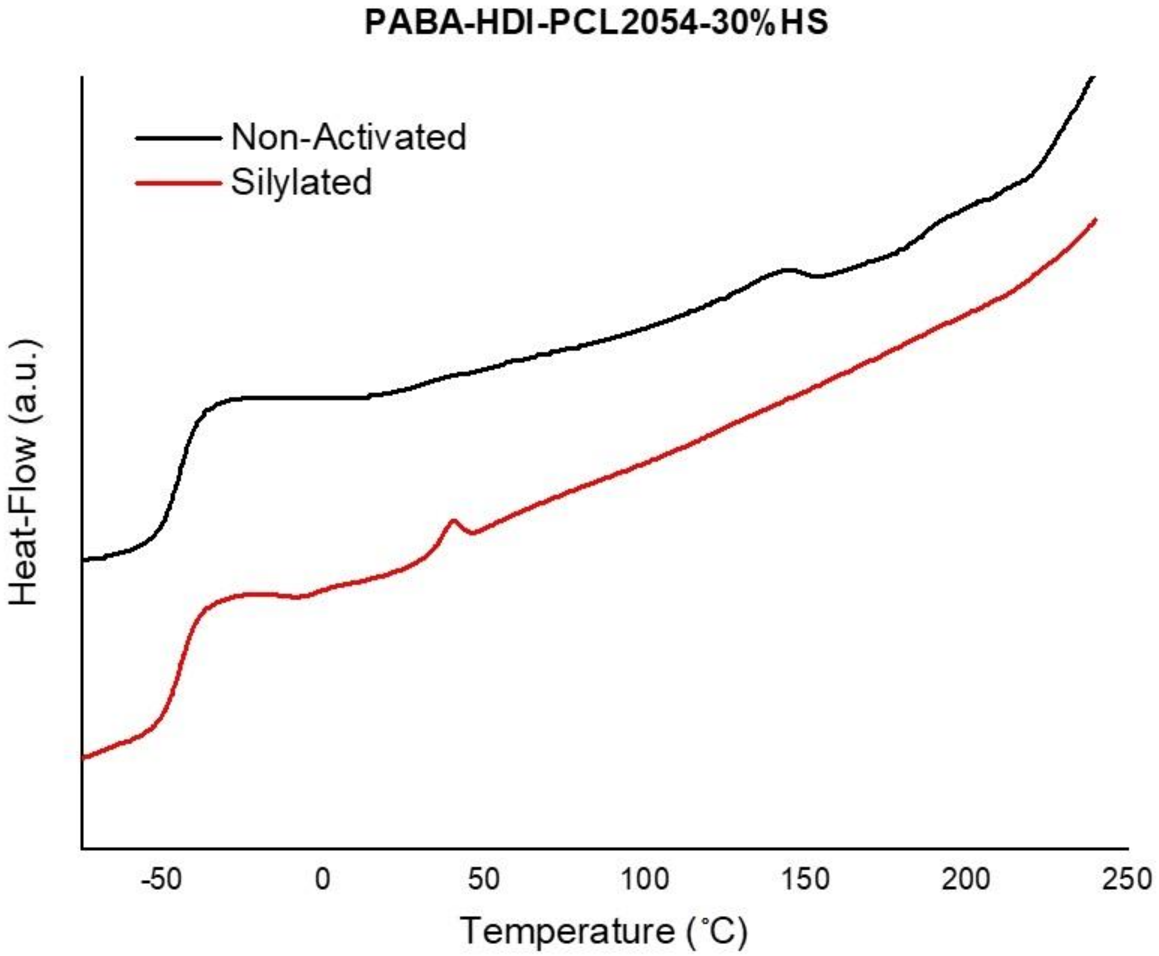

Figure S33. DSC curves for PCL2054-PABA-HDI 30%HS prepared with (red curve) or without silylation (black curve).
